# Chaga Mushroom Triterpenoids Inhibit Dihydrofolate Reductase and Act Synergistically with Conventional Therapies in Breast Cancer

**DOI:** 10.3390/biom14111454

**Published:** 2024-11-17

**Authors:** Junbiao Wang, Daniela Beghelli, Augusto Amici, Stefania Sut, Stefano Dall’Acqua, Giulio Lupidi, Diego Dal Ben, Onelia Bistoni, Daniele Tomassoni, Barbara Belletti, Sanaa Musa, Jamal Mahajna, Stefania Pucciarelli, Cristina Marchini

**Affiliations:** 1 School of Biosciences and Veterinary Medicine, Via Gentile III da Varano, University of Camerino, 62032 Camerino, Italy; daniela.beghelli@unicam.it (D.B.); augusto.amici@unicam.it (A.A.); daniele.tomassoni@unicam.it (D.T.); cristina.marchini@unicam.it (C.M.); 2 DAFNAE Dipartimento di Agronomia, Animali, Alimenti, Risorse Naturali e Ambiente, University of Padova, 35020 Legnaro, Italy; stefania.sut@unipd.it; 3 DSF Department of Pharmaceutical and Pharmacological Sciences, University of Padova, 35121 Padova, Italy; stefano.dallacqua@unipd.it; 4 School of Pharmacy, University of Camerino, 62032 Camerino, Italy; giulio.lupidi@unicam.it; 5 School of Pharmacy-Chemistry Interdisciplinary Project (CHIP), University of Camerino, 62032 Camerino, Italy; diego.dalben@unicam.it; 6 Rheumatology Unit, Department of Medicine and Surgery, University of Perugia, 06123 Perugia, Italy; onelia.bistoni@ospedale.perugia.it; 7 Molecular Oncology Unit, Centro di Riferimento Oncologico di Aviano (CRO), IRCCS, National Cancer Institute, 33081 Aviano, Italy; bbelletti@cro.it; 8 Natural Compounds and Organic Synthesis, Migal-Galilee Research Institute, Kiryat Shmona 11016, Israel; sanaa@migal.org.il (S.M.); jamalm@migal.org.il (J.M.); 9 Department of Biotechnology, Tel Hai College, Kiryat Shmona 1220800, Israel; 10 Cancer Drug Discovery Program, Migal, Galilee Research Institute, P.O. Box 831, Kiryat Shmona 11016, Israel

**Keywords:** Chaga mushroom (*Inonotus obliquus*), triterpenoids, breast cancer, DHFR, cell cycle regulation

## Abstract

*Inonotus obliquus* (Chaga) is a medicinal mushroom with several pharmacological properties that is used as a tea in traditional Chinese medicine. In this study, Chaga water extract was digested in vitro to mimic the natural processing and absorption of its biocomponents when it is consumed as functional beverage, and its anticancer activities were evaluated in breast cancer (BC) cell lines, representing HER2-positive and triple-negative subtypes. After chemical characterization by liquid chromatography/mass spectrometry (HR-QTOF) analysis, the effect of Chaga biocomponents on cell viability and cell cycle progression was assessed by MTT assay, FACS analysis, and Western blot. Dihydrofolate reductase (DHFR) activity was measured by an enzymatic assay. Four highly bioactive triterpenoids (inotodiol, trametenolic acid, 3-hydroxy-lanosta-8,24-dien-21-al, and betulin) were identified as the main components, able to decrease BC cell viability and block the cell cycle in G0/G1 by inducing the downregulation of cyclin D1, CDK4, cyclin E, and phosphorylated retinoblastoma protein. DHFR was identified as their crucial target. Moreover, bioactive Chaga components exerted a synergistic action with cisplatin and with trastuzumab in SK-BR-3 cells by inhibiting both HER2 and HER1 activation and displayed an immunomodulatory effect. Thus, *Inonotus obliquus* represents a source of triterpenoids that are effective against aggressive BC subtypes and display properties of targeted drugs.

## 1. Introduction

Breast cancer (BC) can be classified into four main molecular subtypes based on the expression of estrogen receptor (ER), progesterone receptor (PR), and human epidermal growth factor receptor 2 (HER2) [1,2]. Among them, HER2-positive and triple-negative (ER-negative, PR-negative, and HER2-negative) BCs are associated with the poorest patient survival [3]. Triple-negative BC has the worst prognosis and cannot benefit from targeted therapies, while HER2-positive BC has aggressive behavior, but it can be treated with HER2-targeted therapies such as trastuzumab. However, HER2-targeted therapies have limitations such as intrinsic or acquired drug resistance [4]. Traditional Chinese medicine (TCM) may represent a source of new anticancer compounds, potentially more effective and less toxic than conventional cancer drugs [5,6]. Chinese people have been using TCM for more than 5000 years, and a wealth of information on natural compounds used for the treatment of a variety of diseases including cancer was recorded in ancient medical books. Fungi (sporophores or fruiting bodies) are traditionally used in Chinese medicine as antitumor remedies [7], but the underlying pharmacological mechanisms are not yet completely understood. Recently, many studies have revealed promising anticancer activities of some mushrooms and their active compounds, but there are still no fungal products approved as anticancer therapeutics [8]. Thus, to better exploit their antitumor potential, it is crucial to understand the mechanisms of action of fungal extracts on cancer cells and identify the molecular targets of specific therapeutic components. *Inonotus obliquus*, commonly known as Chaga due to its irregularly formed sterile conk with a burnt charcoal-like appearance, is an edible mushroom belonging to the Hymenochaetaceae family of Basidiomycetes. The presence of Chaga mushrooms is restricted to cold habitats at latitudes of 45° N–50° N, including North America, Central and Northern Europe, Russia (West Siberia), northeast China, and Japan [9]. Chaga mushroom is a parasitic fungus that grows on the bark of various boreal deciduous angiosperms such as birch (*Betula* spp.) and beech (*Fagus* spp.), and it has been used as a folk remedy to treat various diseases such as cancer, cardiovascular diseases, diabetes, and gastrointestinal disorders since the 12th century [10]. Many bioactive constituents, including triterpenoids, polysaccharides, and polyphenols, have been identified in Chaga extracts, and their antitumor, anti-inflammatory, antioxidant, hypoglycemic, and immunomodulatory properties have been described [9,11,12]. Chaga has been reported to exert cytotoxic effects against various types of cancer, including sarcoma [13], lung adenocarcinoma [14], colon cancer [15], melanoma [16], and hepatocellular carcinoma [17]. In this study, we investigated the anti-neoplastic action and the underlying molecular mechanisms of *Inonotus obliquus* extracts against HER2-positive and triple-negative BC. The traditional preparation to consume this fungus is a functional beverage (tea) obtained by the infusion with water of pulverized fungal material to obtain a Chaga water extract. In our work, Chaga water extracts were first treated to simulate human digestion. Bioactive triterpenoids were identified as the main components. Digested Chaga extract was able to block the cell cycle in G0/G1 and exert a synergistic action when administered in combination with trastuzumab in SK-BR-3 (ER-/PR-/HER2+) cells and with cisplatin in both SK-BR-3 and MDA-MB-231 (ER-/PR-/HER2-) cells. 

Dihydrofolate reductase (DHFR), a key enzyme in the amino acids, purines and nucleotide biosynthesis, was identified as a crucial target of *Inonotus obliquus* extract components. Betulinic acid (3β, hydroxyl-lup-20(29)-en-28-oic acid, BA), a pentacyclic triterpene, which is formed by the oxidation of the triterpenoid betulin, one of the major components of digested Chaga extract, was found to affect DHFR activity in both the SK-BR-3 and MDA-MB-231 cell lines.

## 2. Materials and Methods

### 2.1. Inonotus Obliquus Extract

The dried Chaga mushroom (*Inonotus obliquus*) used in this study was purchased in Changchun, Jilin province, China. To obtain an aqueous extract, 10 g of Chaga mushroom was powdered and heated in water at 65 °C for 2 and a half hours with continuous stirring, imitating the traditional preparation system. Subsequently, the solution was filtered, frozen at −20 °C, and eventually lyophilized. The next step involved the in vitro digestion of the lyophilized powder. In vitro digestion helps in understanding and predicting the behavior of food components in the gastrointestinal tract, mimicking what happens in our bodies every time we ingest food or liquids. The simulated digestion method used in this study was described by Minekus et al. and includes the oral, gastric, and small intestinal phases [18]. This method tries to mimic physiological conditions in vivo, considering the presence of digestive enzymes and their concentrations, pH, digestion time, and salt concentrations. The digested solution was then dialyzed using a membrane with a 3500 Da cut-off and placed against water for 12 h to simulate small intestinal absorption. At the end of the incubation process, two solutions were obtained: the one flowing outside dialysis tubing represented the serum-available portion, or in other words, the one absorbed (IN), while the other represented the colon-available solution, which is the non-absorbable sample (OUT). Both solutions, IN and OUT, corresponding to MW < 3500 Da and a MW > 3500 Da extracts of Chaga mushroom, respectively, were collected and lyophilized for further analysis.

### 2.2. Chaga Chemical Characterization 

The analysis of Chaga samples was performed by combining the data from High-Resolution Quadrupole Time of Flight (HR-QTOF) analysis for qualitative approaches and two different liquid-chromatography–mass-spectrometry (LC-MS) methods: one using Electrospray Ion Source (ESI), for the quantification of hydrophilic constituents as sugar derivatives and phenolics, and one using the liquid chromatography atmospheric pressure chemical ionization (LC-APCI) method for the analysis of triterpene and inotodiol related compounds. To perform a qualitative analysis of phytocomplex by LC-QTOF and by LC-ESI-Ion trap, lyophilized samples (100 mg) were suspended in methanol and sonicated for 5 min (15 mL) and then centrifuged. Liquids were used for the analysis. LC-MS analysis was performed in different conditions, and several compounds were identified combining the QTOF-MS and ion trap multiple-stage mass spectrometry (MSn) approaches. Furthermore, ESI and APCI sources have been used to obtain the maximum opportunity to detect and identify constituents. To perform an analysis of terpenoids by LC-APCI-MS, 100 mg of powder was weighed in a flask, and 5 mL of ethyl acetate was added. Flask was sonicated for 10 min and centrifuged for 5 min. Supernatant was removed and collected separately, and the procedure was repeated three times. At the end, the supernatant was pooled, and the ethyl acetate fraction was dried under vacuum with a rotary evaporator. The residual material from the extraction was extracted again with methanol (5 mL three times), and the supernatant was dried under vacuum. Then, the residual material was transferred to a round-bottom flask and dried under vacuum to obtain a dried powder. Five milligrams of powder was weighed and diluted with 1.5 mL of water and sonicated and prepared for the analysis of polysaccharides. Yields for each step were calculated (Supplementary Table S1). Ethyl acetate extracts and methanol extracts were dissolved in methanol (1 mL) and analyzed by LC-APCI-MS. LC was performed using an Agilent 1260 chromatograph equipped with a 1260 diode array (DAD) and an Agilent/Varian MS-500 ion trap (Santa Clara, CA, USA) as detectors. An SB-Aq C18 4.6 × 50 mm 1.8 μm (Agilent, Santa Clara, CA, USA) column was used as a stationary phase and acetonitrile (A), methanol (B), and 0.1% formic acid (FA) in water (C) were used as mobile phases. The elution gradient was set as follows: from 65/1/34% A/B/C to 70/30/0% A/B/C, 0–4 min; isocratic elution for 4–13 min; re-equilibration with the initial solvent ratio for 3 min. The flow rate was 0.6 mL/min, and the injection volume was 10 μL. At the end of the column, a T connector split the flow rate to the DAD and MS detector. MS spectra were recorded in negative ion mode in 50–2000 Da range using an APCI ion source. The turbo data-dependent scanning (TDDS) function allowed us to obtain the fragmentation of the main ionic species. The identification of compounds was based on the fragmentation spectra, as well as the comparison of the fragmentation pattern with the literature and the injection of reference compounds, when available. The DAD chromatograms were monitored at λ = 350, 330, 280, and 254 nm. MS parameters: corona current 5 Amp, neb gas pressure 45 psi, drying gas pressure 15 psi, vaporizing gas pressure 20 psi, drying gas temperature from 320 to 285 °C in 10 min, vaporizing temperature 350, RF loading 81%, capillary 95, and positive ion mode. To establish the presence and the distribution of molecular weight of polysaccharides, we used LC Size-Exclusion Chromatography (SEC) analysis. Water extracts were filtered, and for the analysis, an Agilent 1100 equipped with an Evaporative Light-Scattering Detector (Sedex LX60) was used. As the stationary phase, a Tosohas G3000 was used, and water 0.1% formic acid was used as the mobile phase. This method allows the separation of polymers, and the standards used were dextran 270 KDa, 12 KDa, and 1 KDa. The water extract of each sample was analyzed. All the samples presented a complex chemical composition with compounds with MW < 1 KDa; thus, in the extraction condition, there was poor or no extraction of large-molecular-weight carbohydrates.

### 2.3. Cell Cultures

Human MDA-MB-231, SK-BR-3, CCD 841 CoN (ATCC CRL-1790), and HEK-293 cells were cultured in Dulbecco’s Modified Essential Medium (DMEM, CORNING, Mediatech, New York, NY, USA) supplemented with 10% fetal bovine serum (FBS, Gibco, Life Technologies, Carlsbad, CA, USA) and 1% penicillin–streptomycin (Gibco, Life Technologies). MCF10A cells were cultured in mammary epithelial cell growth medium (PromoCell, Heidelberg, Germany) and 1% penicillin–streptomycin. Cells were cultured at 37 °C under humidified atmosphere with 5% CO_2_. The HEK-293 cell line comprises immortalized human embryonic kidney cells, and they were used as a non-cancerous control cell line, as well as the MCF10A cells, which are a non-tumorigenic mammary epithelial cell line. CCD 841 CoN cells were isolated from normal human colon tissue, and they can be considered as normal colon epithelial cells. Cell lines were kindly provided by the laboratory of Dr. B. Belletti (Division of Molecular Oncology, CRO of Aviano, IRCCS, National Cancer Institute, Aviano, Italy) and tested for mycoplasma contamination with negative results.

### 2.4. Cell Viability Assay

Cell viability was evaluated by seeding MDA-MB-231 cells (7000 cells/well) or SK-BR-3 cells (1 × 10^4^ cells/well), or HEK-293 cells (1 × 10^4^ cells/well) in 96-well plates using complete medium (DMEM supplemented with 10% FBS and 1% penicillin–streptomycin). The day after, fresh medium containing appropriate concentrations of Chaga extract (non-digested or digested), ranging from 0.1 to 5 mg/mL, was added; to evaluate synergistic effects, Chaga extract was administered in combination with trastuzumab (Herceptin, Genentech, San Francisco, CA, USA) or platinum drugs (cisplatin and its derivative RJY13 [19], kindly provided by Prof. Jamal Mahajna and Prof. Sanaa Musa, Galilee Research Institute, Kiryat Shmona, Israel). Betulinic acid (Sigma Aldrich, St. Louis, MO, USA) was tested in a range from 0 to 60 μM. Cell viability was determined, after 24 h, 48 h or 72 h, using an MTT [3-(4,5-dimethylthiazol-2-yl)-2,5-diphenyl-2H-tetrazolium bromide Sigma Aldrich, St. Louis, MO] assay, which is based on the conversion of MTT to formazan by mitochondrial enzymes [20]. The formazan deposits were dissolved in DMSO, and the absorbance of each well was measured at 540 nm in Multiskan Ascent 96/384 Plate Reader. Each drug concentration was evaluated with six replicates, and the experiments were repeated three times. IC_50_ values were calculated for each of the cell lines tested by fitting the concentration–effect curve data obtained in the three experiments with the sigmoid-Emax model using nonlinear regression, weighted by the reciprocal of the square of the predicted effect.

To evaluate drug interaction, the Bliss Independence model was considered using the equation E (x, y) = Ex + Ey − (Ex ∗ Ey), where E is the fractional effect (between 0 and 1), and x and y are the doses (or concentrations) of drugs in the combination. Observed effects greater than E(x, y) indicated synergistic interactions [21].

### 2.5. Western Blot Analysis

Cells were homogenized in RIPA buffer (0.1% SDS, 1% NP40, 0.5% CHAPS) supplemented with protease inhibitors (Sigma-Aldrich, St. Louis, MO, USA). For Western blot analysis, an equal amount of protein lysates was separated onto Criterion™ TGX™ precast gels (Bio-Rad, Hercules, CA, USA) and transferred to a polyvinylidene difluoride (PVDF) membrane (Millipore, Burlington, MA, USA) using Criterion™ Blotter (Bio-Rad). Membranes were blocked with EveryBlot Blocking Buffer (Bio-Rad, Hercules, CA, USA) and then incubated overnight with primary antibodies at 4 °C. Primary antibodies to Src (cat. #2109s, lot 4), p-Src (cat. #2101s, lot 20), and pHER1 (cat. #3777s, lot 10) were from Cell Signaling Technology (1:1000). Primary antibodies to β-actin (sc-47778, lot #K1607), HER2 (sc-284, lot #I0507), p-HER2 (sc-12352-R, lot #D2512), pRb (Ser780) (rabbit sc-12901), p53 (mouse sc-126), Cyclin E2 (mouse sc-28351), and CDK4 (mouse sc-260) were from Santa Cruz Biotechnology. Primary antibody to Cyclin D1(mouse cc12) and secondary antibodies conjugated with peroxidase were from Sigma-Aldrich (Sigma-Aldrich/Merck, Darmstadt, Germany). Secondary antibody binding was performed at room temperature for 1 h. After TBS-T washing, membranes were incubated with PierceTM ECL Western Blotting Substrate (Thermo Scientific, Boston, MA, USA), and the immunoreactive proteins were detected with ChemiDoc™ XRS-System (Bio-Rad, Hercules, CA, USA). Densitometry analysis was performed through ImageJ software (Version: 2.1.0/1.53C).

### 2.6. Cell Cycle Analysis

A total of 5 × 10^5^ SK-BR-3 and MDA-MB-231 cells per well were seeded onto 6-well tissue culture plates. The day after, fresh medium containing 0.5 mg/mL or 1 mg/mL Chaga extract (MW < 3500 Da) was added. After 24 h incubation, the cells were harvested and fixed with ice-cold 70% ethanol, 1 h at 4 °C. RNA was digested by 1 mg/mL bovine RNase (Sigma) 30 min at 37 °C with shaking. Cells were then labeled with 15 mg/mL propidium iodide (PI) 30 min at 37 °C in the dark. Samples were analyzed by fluorescence activated cell sorting (FACS) (BD FACScalibur™, BD Biosciences, San Jose, CA, USA), and data were elaborated via BD CellQuest software (Becton Dickinson and company, Franklin Lakes, NJ, USA, v 8.7). 

### 2.7. DHFR Enzymatic Assay

SK-BR-3 and MDA-MB-231 cells were plated onto 25 cm^2^ flasks (2 × 10^6^ cells/flask). The day after, cells were treated with 0.5 mg/mL or 1 mg/mL digested Chaga extract (MW < 3500 Da) in DMEM supplemented with 2% FBS (Invitrogen, Carlsbad, CA, USA) for 4–6 h. Cell lysates were obtained using Cell Culture Lysis Reagent (Promega, Madison, WI, USA). DHFR activity was assessed by both spectrophotometric assay and discontinuous HPLC assay. In the spectrophotometric assay, the activity of the DHFR was followed by recording the absorbance with a Shimadzu UV-2450 (UV–vis) spectrophotometer. The enzyme assays were carried out in a quartz cuvette by using similar experimental conditions: 100−200 μL of the cell lysate, NADPH 60−80 μM, and dihydrofolate (DHF) 50 μM. After the mixture was stored for 5 min at 37 °C, DHF was added. The decreasing absorbance at 340 nm, due to the oxidation of NADPH to NADP+, was detected after the DHF was added, and expressed in U mL^−1^ using the equation U mL^−1^ = Δabs/Δt/11.8 × dilution factor, where 11.8 is the mMolar extinction coefficient when NADPH and DHF are simultaneously present in solution. In the discontinuous HPLC enzymatic assay, performed as previously described [22], an Agilent 1100 system was used to detect and quantify NADP^+^, as one of the products of DHFR-catalyzed reaction, in cell lysates. Enzyme activities were normalized by the protein content determined by Bradford assay [23]. Betulinic acid (Merck, Rome, Italy) was prepared as a 2 mM stock solution in 100% methanol and used in the 24 h timespan.

### 2.8. Measurements of Cytokines in Conditioned Medium 

The pro- and anti-inflammatory cytokines IL-1α, IL-1β, IL-2, IL-4, IL -5, IL-6, IL-8, IL-10, IL-12, IL-13, IL-15, IL-17, IL-23, IFNγ, TNF-α, and TNF-β were estimated in the culture-conditioned medium of MDA-MB-231 and SK-BR-3 cells by using multiplex immunoassay (Q-Plex Human Cytokine—Screen 16-plex, Quansys Biosciences, Technogenetics Srl., Milan, Italy), Q-View Imager LS, Q-View software Version 3.11, and following the manufacturer’s instructions. The culture medium was obtained by seeding 0.5 × 10^6^ cells in 6-well plates containing complete growing cell culture medium. On the following day, the medium in each well was replaced with 2 mL serum-free medium, and the cells were incubated with or without 0.5 mg/mL Chaga extract MW < 3500 Da, each condition in triplicate for an additional 24 h. Then, the cell culture conditioned medium was collected and centrifuged to remove all dead cells and debris and stored at −80 °C until further analysis.

### 2.9. Statistical Analysis 

Quantitative data are presented as either means ± SD or means ± SE from three independent experiments. The significance of differences was evaluated with an unpaired Student *t* test when two groups were compared, while one-way ANOVA test followed by Tukey’s or Dunnett’s post-test was used to compare three or more groups. Statistical analysis was carried out with GraphPad Prism 8. 

### 2.10. Molecular Modeling

The crystal structure of the human DHFR (pdb code: 1U72; 1.90 Å resolution) [24] was imported into Molecular Operating Environment (MOE) software (version 2022.02) [25] and added to hydrogen atoms. The orientation of the hydrogen atoms was then optimized with energy minimization using the AMBER14 force field and keeping the coordinates of the heavy atoms fixed. The minimizations were performed by steepest descent steps followed by conjugate gradient minimization until the RMS gradient of the potential energy was less than 0.05 kJ mol^−1^ Å^−1^. The betulinic acid’s molecular structure was docked into the binding site of the DHFR using the MOE docking tool by setting the Alpha Triangle placement method and the Alpha HB scoring function with the generation of 50 docking poses. Each pose was then locally energetically minimized (by keeping fixed the target coordinates—Rigid Receptor refinement protocol) and then rescored with Alpha HB scoring function. 

## 3. Results and Discussion

### 3.1. Chemical Characterization of Chaga Extract’s Components

High-Performance Liquid Chromatography (HPLC) analysis provided insights into the Chaga mushroom’s major components. Among them, phenolic acids, terpenes, and carbohydrates proved to be the most abundant. Some other classes of compounds such as coumarins, iridoids, and chalcones were also detected in smaller amounts. Table 1 summarizes the most abundant species in the Chaga samples, identified before in vitro digestion (non-digested), using an untargeted QTOF-HR-ESI-MS approach.

**Figure 1:**
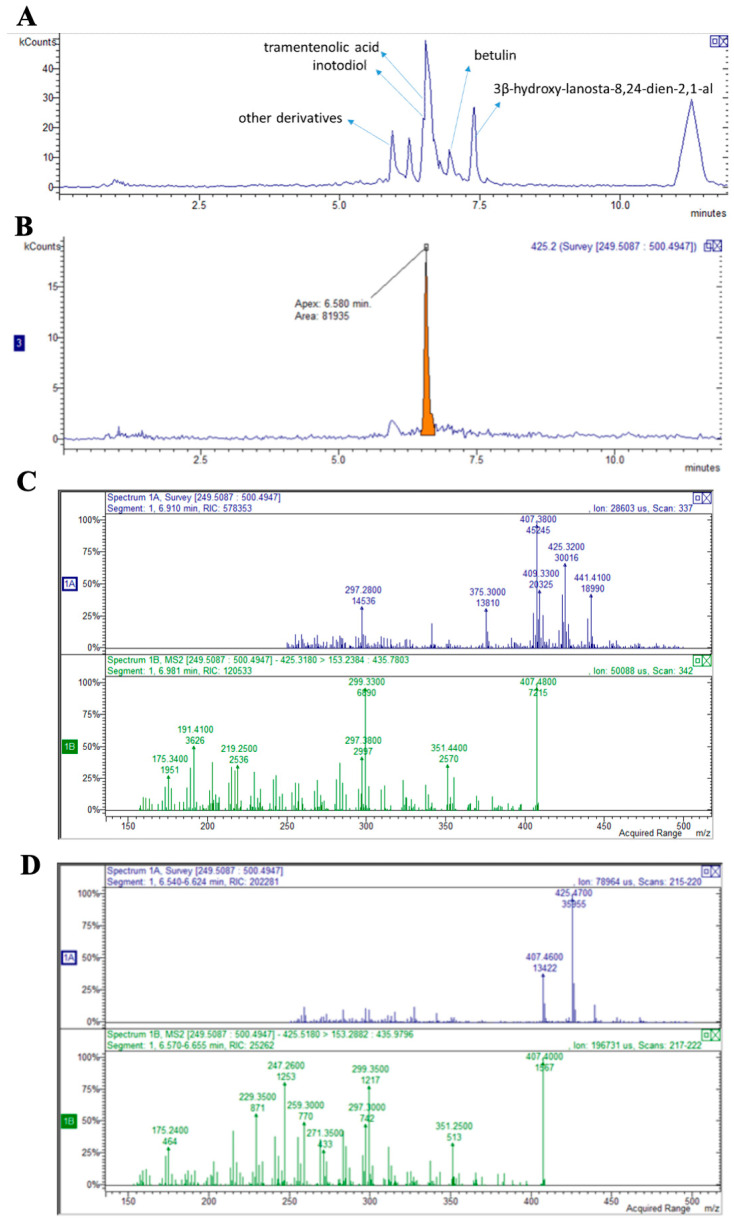
HPLC/MS identification of the terpenoid components of Chaga extract: (**A**) chromatogram with the identified peaks; (**B**) chromatogram recorded in positive mode in which a peak at 6.5 min, with m/z of 425, can be identified as inotodiol; (**C**) MS fragmentation pathway of betulin in positive ion mode showing the species at m/z 425 and fragment ions at m/z 407 and 191; (**D**) MS fragmentation pathway of inotodiol in positive ion mode 425 m/z and fragment ions at m/z 407 and 247.

Under these analytical conditions, the peaks ascribable to inotodiol and its derivatives are detectable but suffer interference due to other compounds and the matrix; thus, a more specific targeted instrumental analysis was adopted to obtain quantitative data about the most important terpenoids in the samples. As an example of terpenoid identification, the chromatogram of the ion at m/z 425 in positive ion mode ascribable to inotodiol (retention time 6.5 min), in agreement with Kim J.H. et al. [26], is reported in Figure 1B, while the MS fragmentation pathway of betulin in positive ion mode showing the species at m/z 425, and fragment ions at m/z 407 and 191, in agreement with the work of Zhang et al. [27], is shown in Figure 1D. The most important identified terpenoids (inotodiol, trametenolic acid, 3-hydroxy-lanosta-8,24-dien-21-al, and betulin) are shown in Figure 1A. Triterpenoids can also be considered the main components of digested Chaga water extract.

### 3.2. Digested Chaga Extract Decreased the Cell Viability of Breast Cancer Cells

To evaluate the anticancer effect of Chaga against HER2-positive BC, SK-BR-3 cell viability was analyzed upon treatment with increasing concentrations of Chaga water extract (non-digested) for 24 h, 48 h, or 72 h. As shown in Supplementary Figure S1A, Chaga treatment decreased SK-BR-3 cell viability in a dose- and time-dependent manner, with IC_50_ values of 0.946 mg/mL after 48 h and 0.671 mg/mL after 72 h. Similar results were obtained by treating triple-negative BC cells with Chaga. Indeed, MDA-MB-231 cell viability was reduced in a dose- and time-dependent fashion by increasing concentrations of Chaga water extract (non-digested) administered for 24 h, 48 h, or 72 h, reaching an IC_50_ value of 0.537 mg/mL at 72 h (Supplementary Figure S1B).

**Figure 2:**
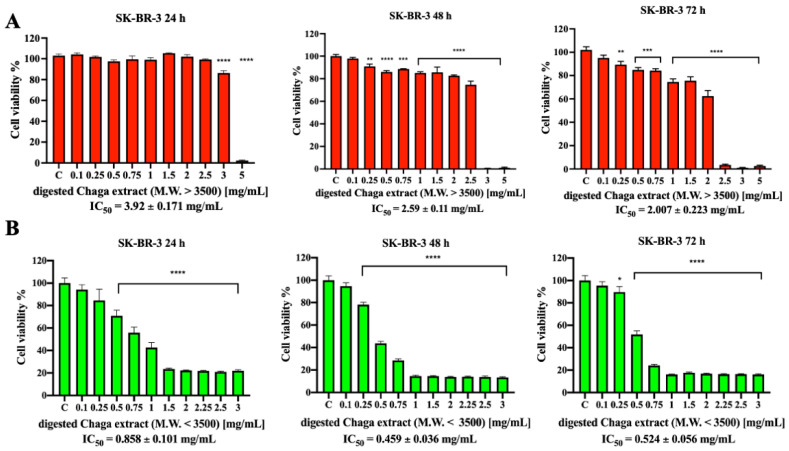
Effect of digested Chaga extract on SK-BR-3 cell viability. SK-BR-3 cells were left untreated (control) or incubated for 24 h, 48 h, or 72 h in the presence of increasing concentrations of the high-molecular-weight fraction (MW > 3500 Da) (**A**) or the low-molecular-weight fraction (MW < 3500 Da) (**B**) of digested Chaga water extract; cell viability was determined by MTT assay. The results are expressed as the percentage of living cells with respect to control. Columns: mean of three separate experiments wherein each treatment was repeated in 6 wells. Bars: SE. * *p* < 0.05, ** *p* < 0.01, *** *p* < 0.001, **** *p* < 0.0001. One-way ANOVA followed by Dunnett’s multiple comparison test.

Considering that *Inonotus obliquus* is used as a functional beverage, Chaga water extracts were treated to simulate human digestion in the gastro-intestinal tract, as described by Minekus et al. [18]. After dialysis, two molecular fractions, a high-molecular-weight fraction (MW > 3500 Da) and a low-molecular-weight fraction (MW < 3500 Da), were obtained, and their anticancer effect was tested on both SK-BR-3 (HER2+) and MDA-MB-231 (HER2-) cells. As shown in Figure 2A, the high-molecular-weight digested Chaga extract (MW > 3500 Da) was able to decrease SK-BR-3 cell viability only at the highest tested concentrations (IC_50_ values of 2.59 mg/mL after 48 h and 2 mg/mL after 72 h). Of note, the low-molecular-weight digested Chaga extract (MW < 3500 Da), instead, induced a strong reduction in SK-BR-3 cells’ viability already after 24 h incubation, showing an IC_50_ value of 0.858 mg/mL, which was further reduced to about 0.46 mg/mL after 48 h (Figure 2B). 

**Figure 3:**
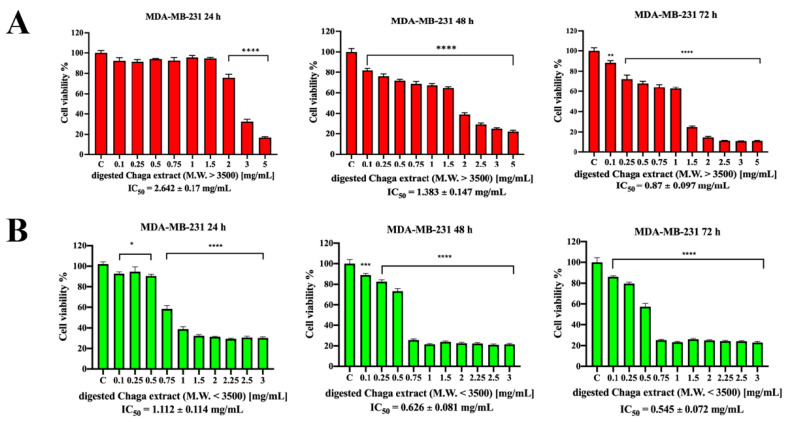
Effect of digested Chaga extract on MDA-MB-231 cell viability. MDA-MB-231 cells were left untreated (control) or incubated for 24 h, 48 h, or 72 h in the presence of increasing concentrations of the high-molecular-weight fraction (MW > 3500 Da) (**A**) or the low-molecular-weight fraction (MW < 3500 Da) (**B**) of digested Chaga water extract; cell viability was determined by MTT assay. The results are expressed as the percentage of living cells with respect to the control. Columns: mean of three separate experiments wherein each treatment was repeated in 6 wells. Bars: SE. * *p* < 0.05, *** *p* < 0.001, **** *p* < 0.0001. One-way ANOVA followed by Dunnett’s multiple comparison test.

Analogously, the high-molecular-weight fraction of Chaga digested water extract (MW > 3500 Da) decreased MDA-MB-231 cell viability in a time-dependent and dose-dependent fashion (Figure 3A), but the most efficient fraction was shown to be the one with MW < 3500 Da. Indeed, the low-molecular-weight digested Chaga extract (MW < 3500 Da) induced a strong reduction in MDA-MB-231 cells’ viability already after 24 h incubation, showing an IC_50_ value of 1.112 mg/mL, which was further reduced to about 0.626 mg/mL after 48 h and to 0.545 mg/mL after 72 h incubation (Figure 3B). Digestion fluid (without Chaga), after dialysis, was also tested on both SK-BR-3 and MDA-MB-231 cells, and the results obtained by MTT assay confirmed that it did not affect cell viability per se (Supplementary Figure S2). However, digested Chaga extract (MW < 3500 Da) also inhibited the viability of HEK-293 cells (IC_50_ = 0.85 ± 0.08 mg/mL at 24 h) and MCF-10A human breast epithelial cells (IC_50_ = 0.086 ± 0.009 mg/mL at 24 h) (Supplementary Figure S3A,B); thus, it seems not to show any apparent selective cytotoxicity to tested cancer cell lines with respect to non-cancer ones. Recently, in contrast to our findings, it has been reported that Chaga extracts were selectively cytotoxic to the MCF-7 breast cancer cell line while sparing the corresponding healthy cell lines (MCF-10A cells). However, when tested at the same concentrations on other non-cancer cell lines, they inhibited their viability [28]. Thus, further investigation is required to assess the selective cytotoxicity of digested Chaga extract to cancer cell lines, extending the study to other healthy cell lines besides HEK-293 and MCF-10A cells. Of note, CCD 841 CoN normal colon epithelial cells appeared much less sensitive to digested Chaga extract (MW < 3500 Da) than the other cells used in this study, showing an IC_50_ of 2.03 ± 0.72 mg/mL after 72 h incubation (Supplementary Figure S3C).

### 3.3. Digested Chaga Extract Induced G0/G1 Cell Cycle Arrest in Breast Cancer Cells

**Figure 4:**
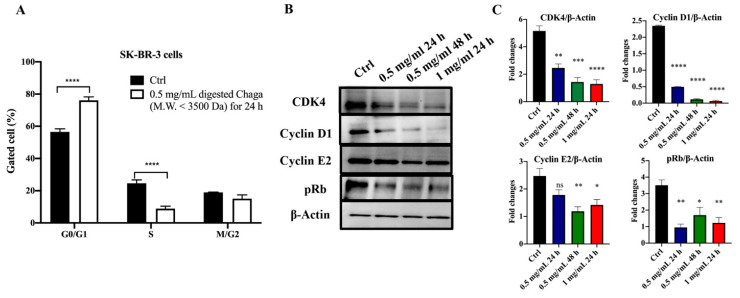
Digested Chaga extract (MW < 3500 Da) induced cell cycle arrest in the G0/G1 phase in SK-BR-3 cells. (**A**) Histograms showing the percentage of SK-BR-3 cells in the G0/G1, S, and G2/M phases in control condition or following 24 h treatment with 0.5 mg/mL of digested Chaga extract (MW < 3500 Da) as assessed by FACS cell cycle analysis. Data are presented as the mean ± SD of three repeats. **** *p* < 0.0001 vs. control. ANOVA followed by Sidak’s multiple comparison test. (**B**) Representative Western blotting showing the expression of CDK4, cyclin D1, cyclin E2, phosphorylated Rb protein (Ser 780), and β-actin (loading control) in SK-BR-3 cells left untreated (Ctrl) or treated with 0.5 mg/mL or 1 mg/mL of digested Chaga extract (MW < 3500 Da) for 24 h or 48 h. Twenty micrograms of proteins/well were loaded. (**C**) Densitometric analysis of each assessed protein. Data are presented as the mean ± SE of three repeats. * *p* < 0.05, ** *p* < 0.01, *** *p* < 0.001, **** *p* < 0.0001. ns: not significant. One-way ANOVA followed by Dunnet’s multiple comparison test.

Next, we investigated the effect of digested Chaga extract (MW < 3500 Da) on SK-BR-3 cells’ distribution in the cell cycle phases by flow cytometry analysis. As shown in Figure 4A, the treatment of SK-BR-3 cells with 0.5 mg/mL of digested Chaga extract (MW < 3500 Da) for 24 h resulted in a higher number of cells in the G0/G1 phase (76.1 ± 1.76%, on average) compared to the control (56.5 ± 1.53%, on average). This increase was coupled with a decreased percentage of digested Chaga-treated SK-BR-3 cells in the S phase (8.85 ± 1.29%, on average) with respect to untreated control cells (24.6 ± 1.68%, on average). Similar results were obtained by treating the SK-BR-3 cells with 1 mg/mL of digested Chaga extract (MW < 3500 Da) for 24 h (Supplementary Table S2). These results suggest that digested Chaga extract induced a G0/G1 arrest of SK-BR-3 cells. To analyze the underlying biochemical mechanisms involved in the G0/G1 cell cycle arrest, we investigated the levels of G0/G1 regulatory cyclins and cyclin-dependent kinases (CDKs) in SK-BR-3 cells treated with digested Chaga extract (MW < 3500 Da) by Western blot (Figure 4, panels B, C). Complexes containing cyclins D and E, the regulatory units, and CDK2, CDK4, or CDK6, the catalytic units, play important roles in the progression of cells through the G0/G1 phase of the cell cycle. Indeed, CDK4/cyclin D complexes phosphorylate retinoblastoma protein (pRb) in mid G1, while CDK2/cyclin E complexes phosphorylate pRb at the G1-to-S transition. The status of pRb phosphorylation is crucial for E2F activity because only the hypophosphorylated form of pRb is associated with E2F transcription factors, which are key regulators of genes required for cell cycle progression. In other words, pRb has a growth-suppressive role (it is active) only when hypophosphorylated, whereas the hyperphosphorylation of Rb protein by cyclins/CDKs results in its inactivation, causing the release of the transcription factor E2F and thus determining cell proliferation [29]. Western blot results showed a significant time-dependent decrease in the levels of cyclin D1, cyclin E2, and CDK4 in SK-BR-3 cells treated with 0.5 mg/mL digested Chaga extract (MW < 3500 Da) for 24 h and 48 h. A further reduction in the levels of the analyzed proteins was observed by increasing the Chaga concentration to 1 mg/mL. Of note, Rb protein was hyperphosphorylated (Ser 780) in untreated cells, whereas digested Chaga extract was effective at reactivating Rb function by decreasing protein phosphorylation in a dose- and-time dependent way, confirming the ability of digested Chaga extract to block the cell cycle progression (Figure 4, panels B, C).

**Figure 5:**
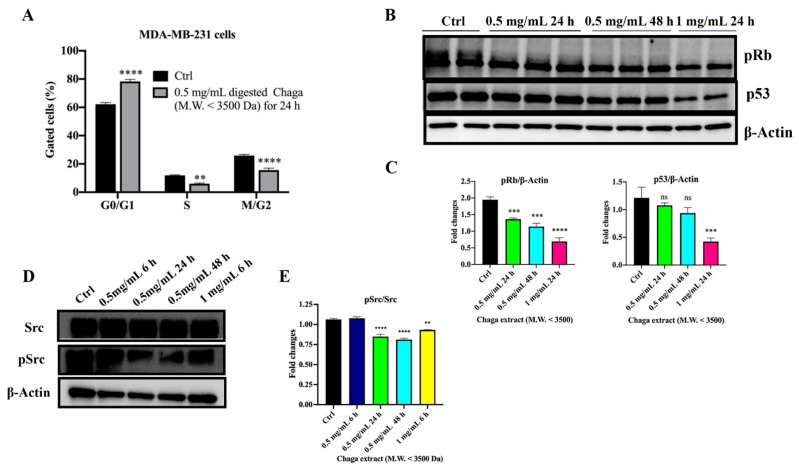
Digested Chaga extract (MW < 3500 Da) induced cell cycle arrest in the G0/G1 phase in MDA-MB-231 cells. (**A**) Histograms showing the percentage of MDA-MB-231 cells in the G0/G1, S, and G2/M phases, in the control condition or following 24 h treatment with 0.5 mg/mL of digested Chaga extract (MW < 3500 Da) assessed by FACS cell cycle analysis. Data are presented as the mean ± SD of three repeats. ** *p* < 0.01, **** *p* < 0.0001 vs. control. ANOVA followed by Sidak’s multiple comparison test. (**B**) Representative Western blotting showing the expression of phosphorylated Rb protein (Ser 780), p53, and β-actin (loading control) in MDA-MB-231 cells, left untreated (Ctrl) or treated with 0.5 mg/mL or 1 mg/mL of digested Chaga extract (MW < 3500 Da) for 24 h or 48 h. Twenty micrograms of proteins/well were loaded. (**C**) Densitometric quantifications of pRb and p53 expression, normalized on β-actin, are shown; data are presented as the mean ± SD of three repeats. *** *p* < 0.001, **** *p* < 0.0001. ns: not significant. One-way Anova, followed by Dunnet’s multiple comparison test. (**D**) Representative Western blotting showing the expression of phosphorylated Src protein (pSrc), Src, and β-actin (loading control) in MDA-MB-231 cells left untreated (Ctrl) or treated with digested Chaga extract (MW < 3500 Da) at the indicated time and concentrations. (**E**) Densitometric quantifications of pSrc/Src from three independent experiments are shown; data are presented as the mean ± SE of three repeats. ** *p* < 0.01, **** *p* < 0.0001. One-way ANOVA, followed by Dunnet’s multiple comparison test.

Treatment with 0.5 mg/mL digested Chaga extract (MW < 3500 Da) for 24 h resulted also in the G0/G1 arrest of MDA-MB-231 cells. Indeed, the percentage of MDA-MB-231 cells in the G0/G1 phase upon Chaga treatment was 78.3 ± 2.12%, on average, whereas it was 62.2 ± 1.63% in the control condition. This increase was associated with the decreased percentage of treated cells in the S phase and in the G2 phase. In particular, the percentage of MDA-MB-231 cells in the S phase was almost double in the control condition (11.88 ± 0.51%) with respect to cells treated with digested Chaga (5.98 ± 0.7%), while 25.9 ± 1.14% of control cells were in the G2 phase versus 15.6 ± 1.9% of digested Chaga-extract-treated MDA-MB-231 cells (Figure 5A). Thus, the expression of two key player proteins involved in the control of cell cycle progression, p53 protein and phosphorylated pRb, was analyzed by Western blotting in MDA-MB-231 cells untreated or treated with 0.5 mg/mL of digested Chaga extract (MW < 3500 Da) for 24 h or 48 h or with 1 mg/mL for 24 h. As shown in Figure 5 (panels B, C), Rb protein was hyperphosphorylated (Ser 780) in untreated cells, whereas digested Chaga extract was effective at reactivating the Rb function by decreasing protein phosphorylation in a dose- and time-dependent way, likely reducing MDA-MB-231 cell proliferation. A similar trend was observed for p53, although a significant decrease in the level of p53 protein was obtained only upon treatment with 1 mg/mL Chaga extract for 24 h (Figure 5, panels B, C). Of note, MDA-MB-231 cells express high levels of a mutant form of p53 (R280K) (mt-p53), which loses the ability to bind responsive elements on DNA, thus becoming defective for oncosuppressor functions. Indeed, accumulating evidence underscores the role of mutant p53 in promoting transformation and metastasis [30]. Thus, the obtained results indicate that digested Chaga extract not only prevented the hyperphosphorylation of pRb in MDA-MB-231 cells, blocking cell cycle progression, but also lowered mt-p53 levels by either stimulating degradation or downregulating the expression of mt-p53. Considering that the tyrosine kinase Src is overexpressed in triple-negative BC and its activation induces tumor cell growth and metastasis [31], we investigated the ability of digested Chaga extract (MW < 3500 Da) to downregulate Src activation. As shown in Figure 5 (panels D, E), Src phosphorylation decreased in a time-dependent way upon the treatment of MDA-MB-231 cells.

### 3.4. Digested Chaga Extract Decreased DHFR Enzymatic Activity in Breast Cancer Cells 

**Figure 6:**
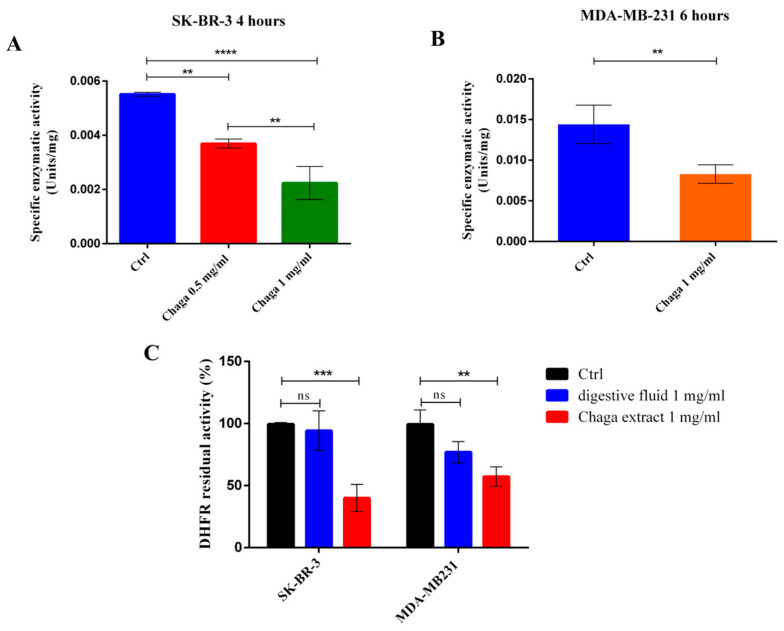
Digested Chaga extract inhibited DHFR enzymatic activity in BC cells. Residual enzymatic activity of DHFR was analyzed in SK-BR-3 (**A**) and MDA-MB-231 (**B**) cells after a 4–6 h treatment with 0.5 mg/mL or 1 mg/mL digested Chaga extract (MW < 3500 Da). (**C**) The residual enzymatic activity of DHFR (as % of the control) is reported in the presence of Chaga extract 1 mg/mL in comparison with the residual activity obtained in the presence of the mixture of digestive fluids used to prepare the Chaga extract at 1 mg/mL. Data are reported as the average of three replicates ± SE, ** *p* ≤ 0.01; *** *p* ≤ 0.001; **** *p* ≤ 0.0001. ns: not significant. One-way ANOVA followed by Tukey’s multiple comparison test.

Due to the central role of the enzyme dihydrofolate reductase (DHFR) in regulating cell viability and proliferation, the effects of Chaga on DHFR activity were also investigated. The enzymatic activity of DHFR was significantly inhibited by Chaga in both SK-BR-3 and MDA-MB-231 cells. In Figure 6, it is possible to observe that the specific activity of DHFR (expressed in enzymatic units/mg of proteins) is strongly affected by low-molecular-weight components of Chaga extract; in particular, 1 mg/mL of digested Chaga in culture medium reduced the residual activity of DHFR to about 50% in both SK-BR-3 and MDA-MB-231 cells. In Figure 6C, the residual enzymatic activity of DHFR (as % of the control) is reported in the presence of Chaga extract 1 mg/mL in comparison with the residual activity obtained in the presence of the mixture of digestive fluids used to prepare the Chaga extract at 1 mg/mL, as described in Materials and Methods. The results show a non-significant effect of digestive fluids in both SK-BR-3 and MDA-MB-231 cells differently from the significant effect exerted by Chaga extract in accordance with the data reported in Figure 6A,B. This result is consistent with the observed effect of the tested Chaga extract on the cell cycle arrest in the G0/G1 phase, as the DHFR enzyme expression levels are known to increase in the G1/S boundary [32]. Furthermore, we can also hypothesize that some of the components of the extract can directly inhibit DHFR enzymatic activity, as observed by pre-incubating the BC cell lysates with 0.1 mg/mL of a low-molecular-weight fraction (MW < 3500 Da) of digested Chaga water extract directly in the reaction mixture (Supplementary Figure S6).

### 3.5. Digested Chaga Displayed a Synergistic Activity When Combined with Trastuzumab and Cisplatin in Breast Cancer Cells

**Figure 7:**
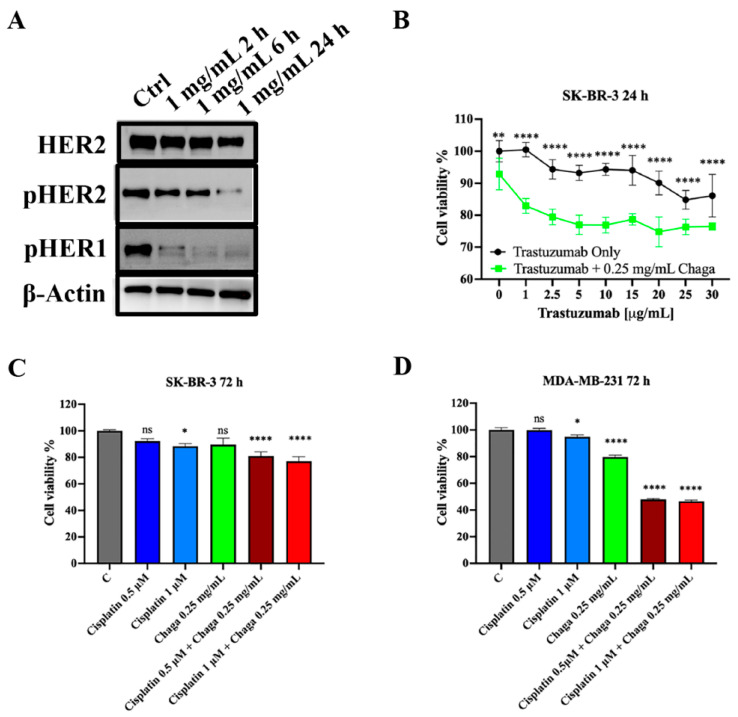
Digested Chaga extract impaired HER2 activation and acted synergistically with trastuzumab and cisplatin. (**A**) Representative Western blotting showing the expression of HER2, phospho-HER2, phospho-HER1, and β-actin (loading control) in SK-BR-3 cells, left untreated (Ctrl) or treated with 1 mg/mL of digested Chaga extract (MW < 3500 Da) for 2 h, 6 h, or 24 h. Twenty micrograms of proteins/well were loaded. (**B**) SK-BR-3 cells were plated onto 96-well plates, treated with increasing concentrations of trastuzumab alone or in combination with a fixed, sub-toxic concentration (0.25 mg/mL) of digested Chaga extract (MW < 3500 Da); cell viability was determined by MTT assay. (**C**) SK-BR-3 cells and (**D**) MDA-MB-231 cells were treated with the indicated concentrations of cisplatin alone or in combination with 0.25 mg/mL digested Chaga extract (MW < 3500 Da); cell viability was determined by MTT assay. Drug interaction was evaluated by the Bliss Independence model; the observed effects of the drug combination indicated synergistic interaction (calculations are reported in Table S3). Bars: SE. * *p* < 0.05, ** *p* < 0.01, **** *p* < 0.0001. ns: not significant. One-way ANOVA followed by Dunnett’s multiple comparison test. Data show a representative of three independent experiments.

The molecular mechanisms underlying the Chaga anticancer effect were further investigated in SK-BR-3 cells, with a focus on the impact of digested Chaga extract (MW < 3500 Da) on HER2 expression and activation. Considering that growth factor receptors do not act as single proteins but as homo- or hetero-dimers, the level of activation of HER1 was also evaluated. Chaga was able to significantly decrease in a time-dependent manner the activation of both HER2 and HER1, as indicated by the downregulation of their phosphorylated forms (Figure 7A). This result provided the rationale for evaluating the synergistic anticancer effect of digested Chaga extract and trastuzumab, a well-established HER2 directed monoclonal antibody used as targeted therapy in HER2-positive BC patients. As shown in Figure 7B, the combination of 0.25 mg/mL digested Chaga extract (MW < 3500 Da) with trastuzumab shows a synergistic antitumor activity, significantly reducing SK-BR-3 cell viability with respect to trastuzumab treatment alone after 24 h incubation. Interestingly, Chaga was also able to synergistically enhance the action of cisplatin, a conventional chemotherapeutic agent, in both SK-BR-3 and MDA-MB-231 cells (Figure 7C,D, Supplementary Figure S3). Indeed, the cell viability of SK-BR-3 and MDA-MB-231 cells decreased significantly only upon treatment with the combination of 0.25 mg/mL Chaga and 0.5 µM or 1 µM cisplatin, whereas cisplatin alone was not effective at all when given at 0.5 µM or exerted a low inhibition at 1 µM. Similar results were obtained using the cisplatin derivative RJY13 [19], a platinum (IV)–fatty acid conjugate ((cis, cis, trans-[diamminedichloro-bisoctanoatoplatinum (IV)]) (Supplementary Figures S4 and S5). 

Drug interaction was evaluated by Bliss Independence model, which provided evidence that the observed effects of drug combinations were greater than the sum of the individual effects of each drug, indicating synergistic interactions (Table S3). The synergistic effect between cisplatin and Chaga extract observed in MDA-MB-231 cells is particularly important, considering that platinum-containing regimens are recommended in the treatment of early and advanced triple-negative BC [33]. Thus, the combination of cisplatin with Chaga extract has the potential to improve chemotherapy efficacy, to decrease the risk of cancer treatment resistance, and to reduce drug side effects thanks to the lowering of its doses [34].

**Figure 8:**
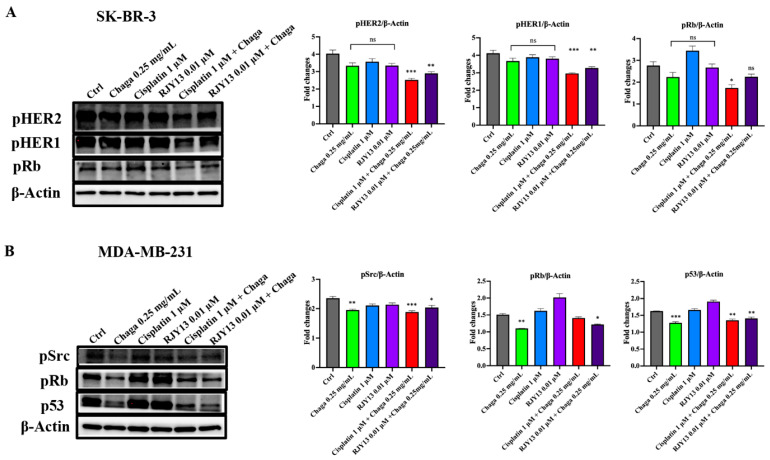
Oncogenic pathways in SK-BR-3 and MDA-MB-231 cells treated with Chaga extract in combination with platinum-based chemotherapeutics. (**A**) Left panel: representative Western blotting showing the expression of phosphorylated (p) HER1, pHER2, pRb protein (Ser 780), and β-actin (loading control) in SK-BR-3 cells left untreated (Ctrl) or treated with 0.25 mg/mL of digested Chaga extract (MW < 3500 Da) alone, 1µM cisplatin or its derivative 0.01 µM RJY13 alone, or their combination for 72 h. Twenty micrograms of proteins/well were loaded. Right panel: densitometric quantifications of pHER1, pHER2, and pRb expression, normalized on β-actin, are shown; data are presented as the mean ± SE of three repeats. (**B**) Left panel: representative Western blotting showing the expression of phosphorylated (p) Src, pRb protein (Ser 780), p53, and β-actin (loading control) in MDA-MB-231 cells left untreated (Ctrl) or treated with 0.25 mg/mL of digested Chaga extract (MW < 3500 Da) alone, 1 µM cisplatin or its derivative 0.01 µM RJY13 alone, or their combination for 72 h. Twenty micrograms of proteins/well were loaded. Right panel: densitometric quantifications of pSrc, pRb, and p53 expression, normalized on β-actin, are shown; data are presented as the mean ± SE of three repeats. * *p* < 0.05, ** *p* < 0.01, *** *p* < 0.001. ns: not significant. One-way ANOVA, followed by Dunnet’s multiple comparison test.

In SK-BR-3 cells, the synergistic action between Chaga and platinum-based compounds was associated with a statistically significant reduction in the level of phosphorylated HER1 and phosphorylated HER2, as well as with a lower level of phosphorylated Rb (Figure 8A). In MDA-MB-231 cells, sub-toxic concentrations of cisplatin alone (1 µM) or its RJY13 derivative (0.01 µM) were not able to affect the activation of Src, the hyperphosphorylation of Rb, or the expression of mt-p53, but the expression of these key molecules governing cell proliferation and survival significantly decreased when platinum drugs were administered in combination with Chaga extract (Figure 8B). However, in this case, the combination of cisplatin and Chaga extract was no more effective than Chaga extract alone.

### 3.6. Betulinic Acid Exerts Anticancer Effects on SK-BR-3 and MDA-MB-231 Cancer Cells and Impairs DHFR Enzymatic Activity 

**Figure 9:**
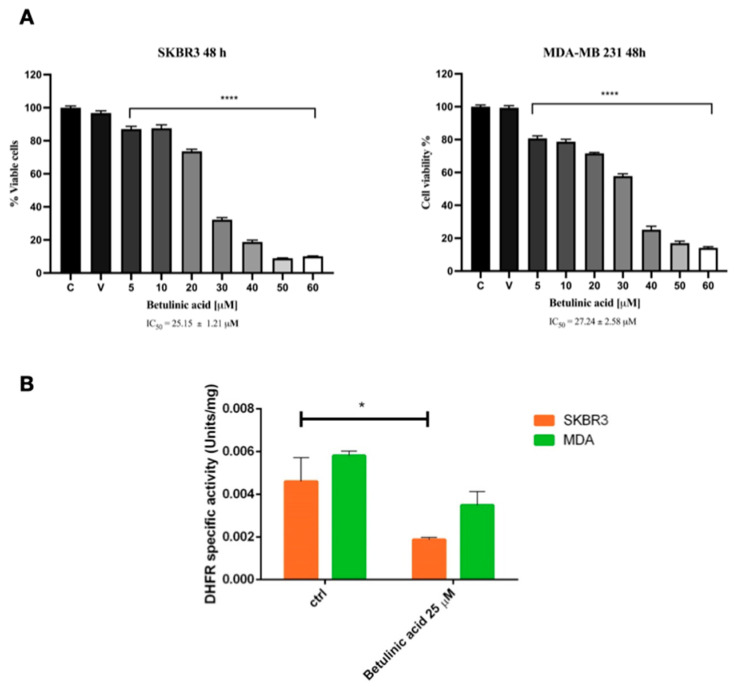
Sensitivity of breast cancer cells SK-BR-3 and MDA-MB-231 to betulinic acid in terms of (**A**) cell viability and (**B**) DHFR enzymatic activity. Data are reported as the average of three replicates ± SE, * *p* < 0.05, **** *p* < 0.0001. One-way ANOVA followed by Tukey’s multiple comparison test.

The terpenoid betulinic acid was administered to the SK-BR-3 and MDA-MB-231 cancer cell lines to evaluate the anticancer effect over a 48-h time course at concentrations ranging from 5 to 60 µM. As shown in Figure 9A, in the two cell lines, the IC_50_ was very similar and was shown to be 25.15 ± 1.21 µM in SK-BR-3 and 27.24 ± 2.58 µM in MDA-MB-231 cells. These values of IC_50_ are in good agreement with data reported elsewhere [35]. 

Based on the IC_50_ values, SK-BR-3 and MDA-MB-231cancer cells were treated with 25 µM of betulinic acid, and the DHFR enzymatic activity was assayed in the cell lysates, as described in the Materials Methods, under saturating concentrations of both NADPH and dihydrofolic acid.

In Figure 9B, it is possible to observe how the SKBR3 and MDA-MB-231 cancer cells respond to the treatment with 25 µM betulinic acid by reducing the enzymatic activity of DHFR of about 40–50% in both cell lines.

A docking study was performed to simulate and analyze the binding mode of the betulinic acid molecule within the DHFR binding cavity. Hence, the crystal structure of the human enzyme in complex with methotrexate and the NADPH co-factor was downloaded from the PDB database (pdb code: 1U72; 1.90 Å resolution) [24] and added to hydrogen atoms that were energetically minimized within the Molecular Operating Environment (MOE) [25]. Docking experiments were also performed within MOE, with a Rigid Receptor refinement protocol aimed at energetically minimizing the generated docking poses. 

**Figure 10:**
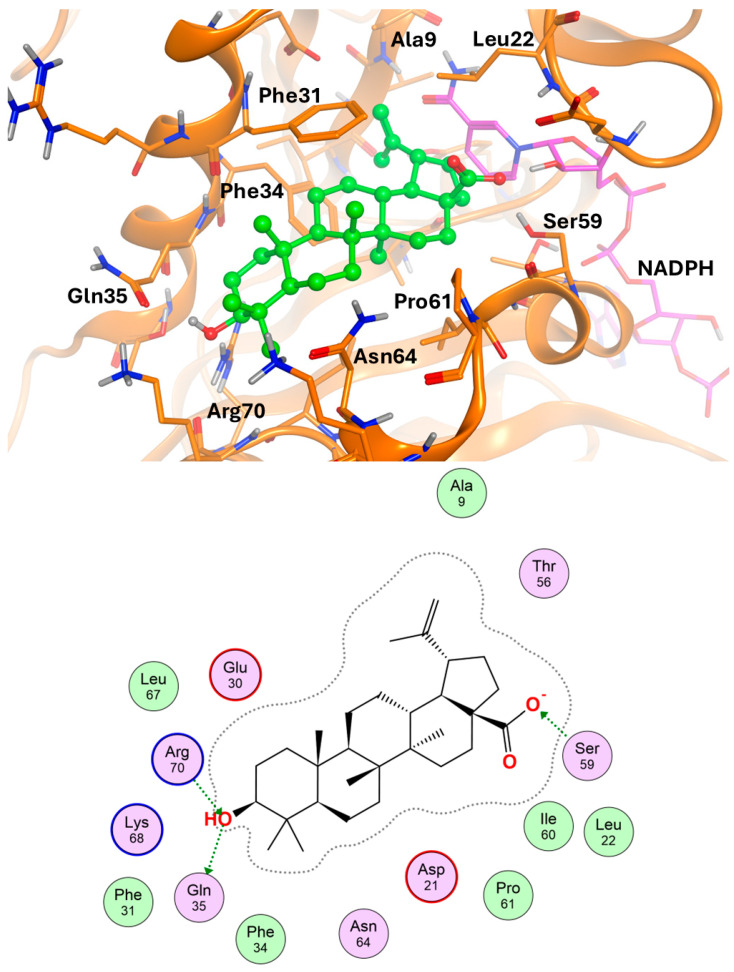
(**Above**): simulated binding mode of the betulinic acid (green) at the binding cavity of the DHFR; the key residues for ligand–target interaction are shown. (**Below**): schematic plot of ligand–target interaction.

The docking result for betulinic acid is shown in Figure 10. The top-score docking pose presents the ligand inserted in the binding side with its isopropenyl group in the depth of the cavity. The interaction with the enzyme is largely non-polar, given by contacts between the ligand scaffold and hydrophobic residues like Phe31, Phe34, and Pro61. Polar interactions are present between the carboxyl function of betulinic acid and Ser 59 and between the hydroxyl group of the same molecule and the DHFR residues Gln35 and Arg70. 

### 3.7. Digested Chaga Displayed Immunomodulatory Properties in Breast Cancer Cells 

**Figure 11:**
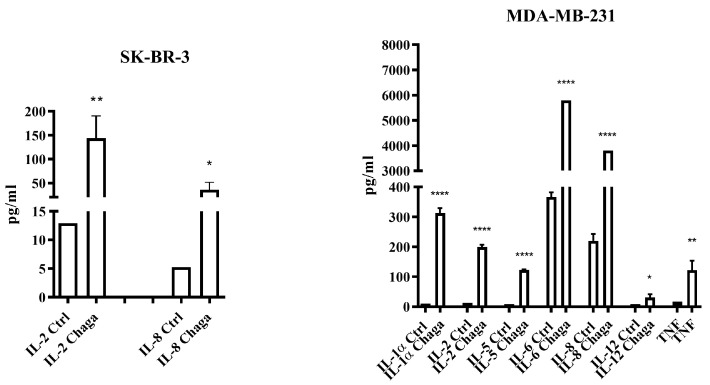
Digested Chaga extract (MW < 3500 Da) induced cytokine release in the culture medium of both SK-BR-3 and MDA-MB-231 cultures but differently according to the cell line. Cytokines were quantified by using a multiplex immunoassay, as described in Materials and Methods section. Data were analyzed by Student’s *t*-test. * *p* < 0.05, ** *p* < 0.01, **** *p* < 0.0001, with respect to the corresponding Ctrl supernatant cytokine concentration.

To evaluate the ability of digested Chaga extract (MW < 3500 Da) to modulate the pro- and anti-inflammatory cytokine secretion by SK-BR-3 and MDA-MB-231 cells, the concentration of a panel of 16 cytokines (IL-1α, IL-1β, IL-2, 4, IL-5, IL-6, IL-8, IL-10, IL-12, IL-13, IL-15, IL-17, IL-23, IFNγ, TNF-α, and TNF-β) was measured in culture supernatants by a multiplex ELISA assay. IL-2 and IL-8 were the only cytokines detectable in the culture medium of 0.5 mg/mL Chaga-treated SK-BR-3 cells, whereas all the other cytokines were below the detection level. The treatment of MDA-MB-231 cells with 0.5 mg/mL Chaga induced not only an increase in the levels of IL-2 and IL-8 in the culture medium but also an elevation of IL-1α, IL-5, IL-6, IL-12, and TNFα, as well as IL-6 and IL-8 (both out of kit’s scale in Chaga-treated MDA-MB-231 cells), although these last two cytokines were already detectable in control samples (Figure 11). 

The production of cytokines by different tumor cell lines, although cultivated under identical conditions, can considerably vary [36]. Our results are consistent with the data obtained from Hartman et al. [37], which indicate that the MDA-MB-231 cell line expresses high amounts of IL-6, and both pro- and anti-apoptotic functions of this cytokine have been reported [38]. Although it is known that the aqueous extract of *Inonotus obliquus* exerts anti-inflammatory effects by down-regulating the expression of pro-inflammatory mediators [39], in the present study, depending on the cell line considered, the Chaga-treated cells over-secreted these mediators (IL-1, IL-2, IL-5, IL-6, IL-8, and TNF-α). However, accumulating data have nowadays shown that cytokines play an important role in both induction and protection in BC [40]. On the one hand, the IL-2 induces the expansion of CD4^+^ and CD8^+^ T lymphocytes and thus could reinforce the anticancer immune responses; on the other hand, the role of IL-8 in antitumor immune responses is more controversial. Indeed, although IL-8 has been reported to favor cancer progression and metastases [41], it was also shown to be responsible for recruiting neutrophils and macrophages, which in turn can kill antibody-opsonized cancer cells by a mechanism of cytotoxicity called trogoptosis [42]. Of interest also is the increased level of IL-5 in the supernatant of MDA-MB-231 cells treated with Chaga. High levels of IL-5 were found in tumor interstitial fluid samples and were associated with a worse BC prognosis [43]. Nevertheless, an increased IL-5 production was recently found to be crucial for systemic eosinophil expansion and tumor infiltration in patients with metastatic triple-negative BC responding to immune checkpoint blockade treatment [44]. These results demonstrate that digested Chaga extract has immunomodulatory properties, in agreement with other studies reporting that medicinal mushroom components can modulate the immune system via a variety of molecular processes, including cytokine induction [45]. In particular, Chaga has been found to activate innate immunity, enhancing the phagocytosis of macrophages [46] and the maturation of dendritic cells [47].

## 4. Conclusions

Chaga mushrooms (*Inonotus obliquus*) are commonly used as traditional treatments in Asia due to their diverse pharmacological effects and in some cases are claimed to some anti-tumor effects. Considering that Chaga is usually consumed as a functional beverage (tea), we investigated the anticancer properties of “digested” Chaga water extracts against BC. Triterpenoids were identified as the main components of digested Chaga water extract, which was able to reduce cancer cell viability, to interfere with oncogenic signaling pathways and to induce a cell cycle G0/G1-phase arrest in both triple-negative (MDA-MB-231) and HER2-positive (SK-BR-3) BC cell lines. These data are consistent with previous investigations reporting the anti-tumor potential of triterpenoids, among Chaga phytochemical constituents [8,12]. These effects were associated with immunomodulatory actions and with the inhibition of the enzymatic activity of the enzyme dihydrofolate reductase (DHFR), which has a key role in the de novo synthesis of purines and thymidylate and thus regulates cell viability and proliferation. Moreover, digested Chaga treatment was able to act synergistically with trastuzumab and cisplatin.

In addition, we could identify in the triterpene betulinic acid that originates from betulin, as one of the putative bioactive components of Chaga extract able to impair breast cancer cell viability and inhibit DHFR activity at micromolar concentrations. 

In conclusion, the present study provides evidence that digested Chaga extract is effective against aggressive BC subtypes, targeting key molecules associated with the malignant phenotype, and demonstrates that *Inonotus obliquus* can represent a good source of alternative antitumor drugs or a remedy that, in combination with conventional drugs, may increase their effectiveness or reduce their dosage.

## Figures and Tables

**Table 1:** Qualitative analysis of the compounds detected in Chaga.

RT (min)	m/z	Tentative Identification	Adduct Ion	Chemical Class
4.17	177.0532	4-Methoxycinnamic acid	[M-H]^−^	Phenolic acid
4.51	511.144	3-(4-Hydroxy-3-methoxyphenyl)-1,2-propanediol 2-O-(galloyl-glucoside)	[M-H]^−^	Phenolic acid
3.38	179.0327	Caffeic acid	[M-H]^−^	Phenolic acid
4.68	535.1082	Lyoniresinol 9′-sulfate	[M+Cl]^−^	Phenolic acid
4.4	539.1753	Orientaloside	[M-H]^−^	Phenolic acid
4.61	197.0428	Syringic acid	[M-H]^−^	Phenolic acid
3.88	285.0595	Uralenneoside	[M-H]^−^	Phenolic acid
5.14	735.213	Feruloylquinic acid	[2M-H]^−^	Phenolic acid
3.91	555.1705	Cassitoroside	[M-H]^−^	Phenolic acid
3.25	311.0385	Caftaric acid	[M-H]^−^	Phenolic acid
4.99	649.2119	Egonol gentiobioside	[M-H]^−^	Phenolic acid
4.49	423.1283	Gibberellin A32	[M+FA-H]^−^	Terpene
6.42	533.3084	Ganoderic acid L	[M-H]^−^	Terpene
4.99	737.229	Polyporusterone B/C	[M+FA-H]^−^	Terpene
6.96	549.3419	Protobassic acid	[M+FA-H]^−^	Terpene
7.71	533.3464	Ganoderiol D	[M+FA-H]^−^	Terpene
4.85	509.1291	D-Galactopyranosyl-(1->3)-D-galactopyranosyl-(1->3)-L-arabinose	[M+Cl]^−^	Carbohydrate
4.61	391.1012	Galactopinitol A	[M+Cl]^−^	Carbohydrate
4.27	449.1071	a-L-Arabinofuranosyl-(1->3)-b-D-xylopyranosyl-(1->4)-D-xylose	[M+Cl]^−^	Carbohydrate
3.91	449.1068	a-L-Arabinofuranosyl-(1->3)-[a-L-arabinofuranosyl-(1r5)]-L-arabinose	[M+Cl]^−^	Carbohydrate
4.91	391.1008	Galactopinitol B	[M+Cl]^−^	Carbohydrate
4.51	369.0804	5-Hydroxy-6-methoxycoumarin 7-glucoside	[M-H]^−^	Coumarin
4.17	383.0959	Eleutheroside B1	[M-H]^−^	Coumarin
4.76	537.1604	Lippioside I	[M-H]^−^	Iridoid
4.3	553.1552	Lippioside II	[M-H]^−^	Iridoid
4.91	421.112	2′,4′,3,4,alpha-Pentahydroxydihydrochalcone 3′-C-xyloside	[M-H]^−^	Chalcone
3.83	273.0381	1,3,6-Trihydroxy-5-methoxyxanthone	[M-H]^−^	Xanthone
7.66	505.3156	2-deoxy-20-hydroxy-5alpha-ecdysone 3-acetate	[M-H]^−^	Ecdysteroid
7.34	549.3422	Desglucocoroloside	[M+FA-H]^−^	Cardenolide
4.02	245.0064	Glucaric acid	[M+Cl]^−^	Polyol
3.82	251.0531	Methionyl-Cysteine	[M-H]^−^	Dipeptide

## Data Availability

The data presented in this study are available in this article and Supplementary Material.

## Supplementary Materials

The following supporting information can be downloaded at https://www.mdpi.com/article/10.3390/biom14111454/s1. Table S1: Yields of Chaga extracts with ethyl acetate, methanol, and water. Figure S1: Chaga water extract decreased breast cancer cell viability. Figure S2: Digested fluid (without Chaga) did not affect SK-BR-3 and MDA-MB-231 cell viability. Table S2: Digested Chaga extract (MW < 3500 Da) induced cell cycle arrest in the G0/G1 phase in SK-BR-3 cells. Figure S3: Effect of digested Chaga extract on cell viability of non-cancerous cell lines. Figure S4: Effect of cisplatin treatment on SK-BR-3 and MDA-MB-231 cell viability. Figure S5: Synergistic effect between Chaga and RJY13 (cisplatin derivative) treatment on SK-BR-3 and MDA-MB-231 cell viability. Table S3: Drug interaction evaluated by the Bliss Independence model. Figure S6: Digested Chaga extract inhibited DHFR enzymatic activity in BC cells lysates. The original images of Western blot can be found in Supplementary Materials.
