# Supplementary material for: Chaga Mushroom Triterpenoids Inhibit Dihydrofolate Reductase and Act Synergistically with Conventional Therapies in Breast Cancer
Source: Biomolecules. 2024 Nov 17;14(11):1454. doi: 10.3390/biom14111454 (PMC11591880; doi:10.3390/biom14111454)
Supplement: Supplementary file 1 [file biomolecules-14-01454-s001.zip › biomolecules-3176010-original-images.pdf]

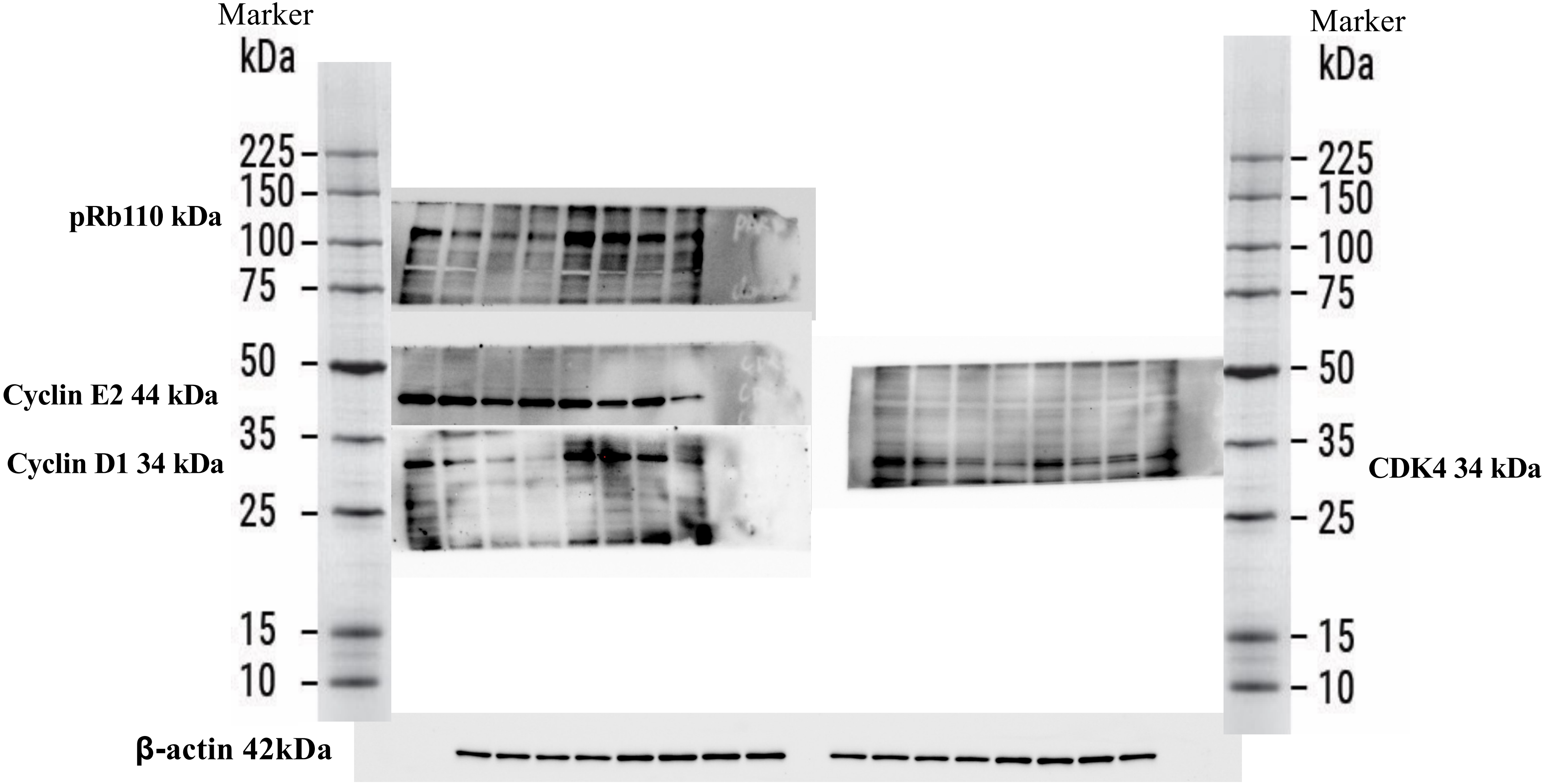

Original western blots from which Figure 4 (Panel B) derived: analysis of the expression levels of CDK4, cyclin D1, cyclin E, phosphorylated Rb protein (Ser 780) and β-actin (loading control) in SK-BR-3 cells. 20 μg of proteins/well were loaded.

	Ctrl	0.5 mg/ml 24h	0.5 mg/ml 48h	1 mg/ml 24h
CDK/β-Actin	5.456	2.744	1.78	1.599
Cyclin D1/β-Actin	2.345	0.487	0.106	0.037
Cyclin E2/β-Actin	2.409	1.718	1.011	1.23
pRb/β-Actin	3.257	0.95	1.804	1.27

Corresponding to Figure 4 B,C. Densitometry Readings/Intensity Ratio

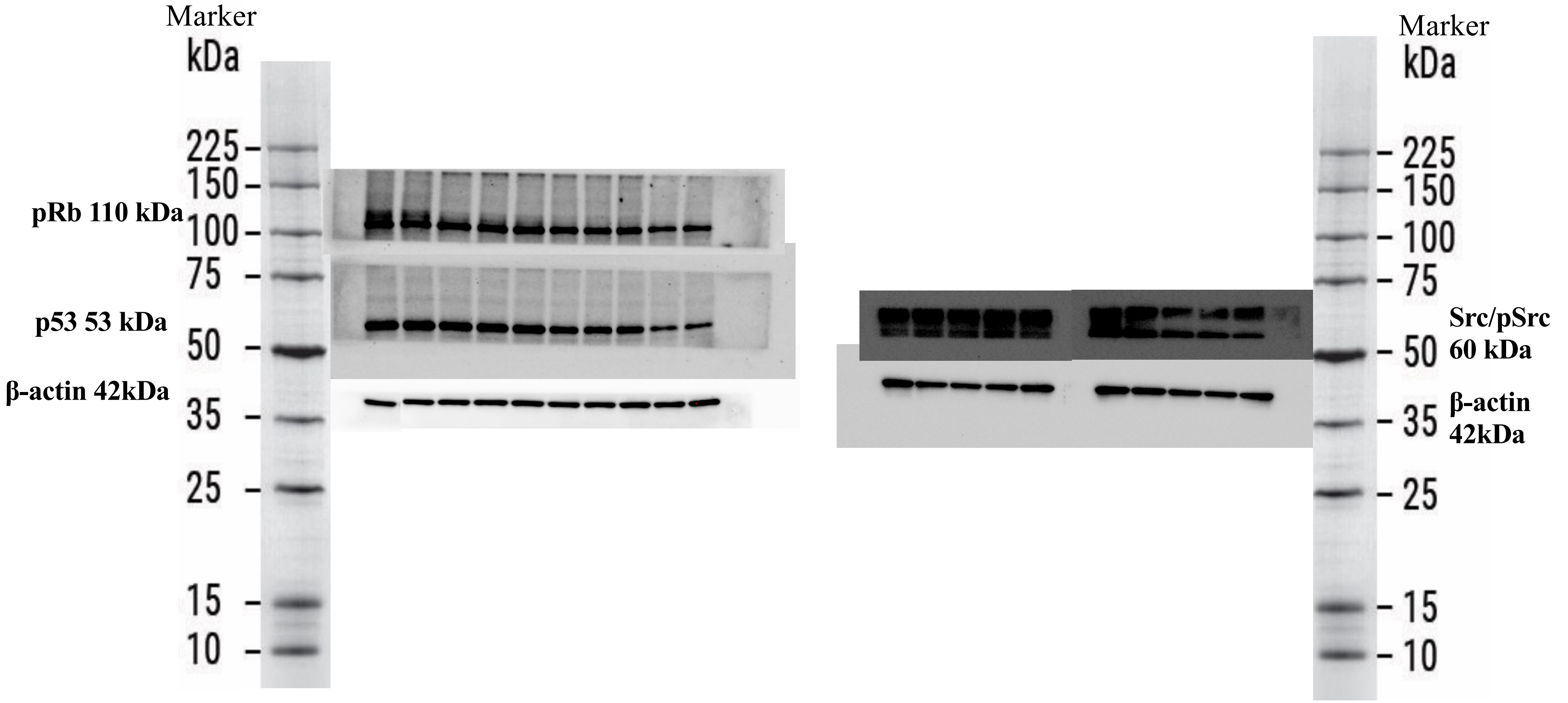

Original western blots from which Figure 5 (Panel B, D) derived: analysis of the expression levels of phosphorylated Rb protein (Ser 780), p53, phosphorylated Src protein (pSrc), Src and β-actin (loading control) in MDA-MB-231 cells, 20 μg of proteins/well were loaded.

	Ctrl	0.5 mg/ml 24h	0.5 mg/ml 48h	1 mg/ml 24h
pRb/β-Actin	1.889	1.358	1.129	0.77
p53/β-Actin	1.349	1.128	0.839	0.377

Corresponding to Figure 5 B,C. Densitometry Readings/Intensity Ratio

	Ctrl	0.5mg/mL 6h	0.5mg/ml 24h	0.5mg/ml 48h	1mg/ml 6h
Src	35588.296	35263.962	35837.276	35287.276	35618.79
pSrc	37401.012	38499.468	30493.66	28479.418	33575.761
β-Actin	16488.69	11657.397	10202.719	11982.983	15381.225
pSrc/Src	1.051	1.092	0.851	0.807	0.943

Corresponding to Figure 5 D,E. Densitometry Readings/Intensity Ratio

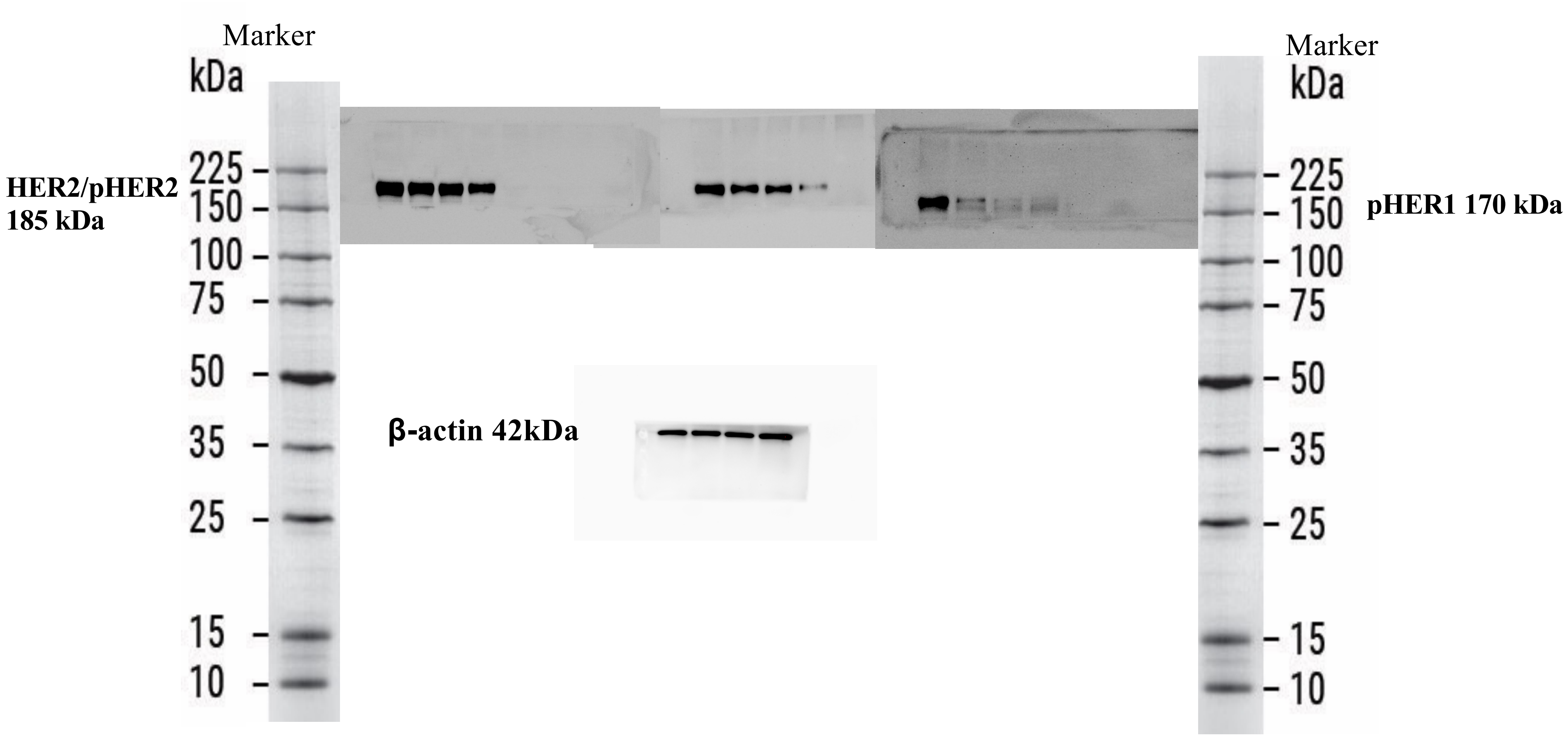

Original western blots from which Figure 7 (Panel A) derived: analysis of the expression levels of HER2, phospho-HER2, phospho-HER1 and β-actin (loading control) in SK-BR-3 cells. 20 µg of proteins/well were loaded.

SKBR3	Ctrl	1mg/ml 2h	1mg/ml 6h	1mg/ml 24h
HER2	35200.551	29831.723	39305.853	30782.158
pHER2	95612.768	16352.116	6749.078	5747.61
pHER1	27396.187	7845.238	4057.702	6253.823
β-actin	23419.832	25865.953	28169.418	35085.53

Corresponding to Figure 7A. Densitometry Readings/Intensity Ratio

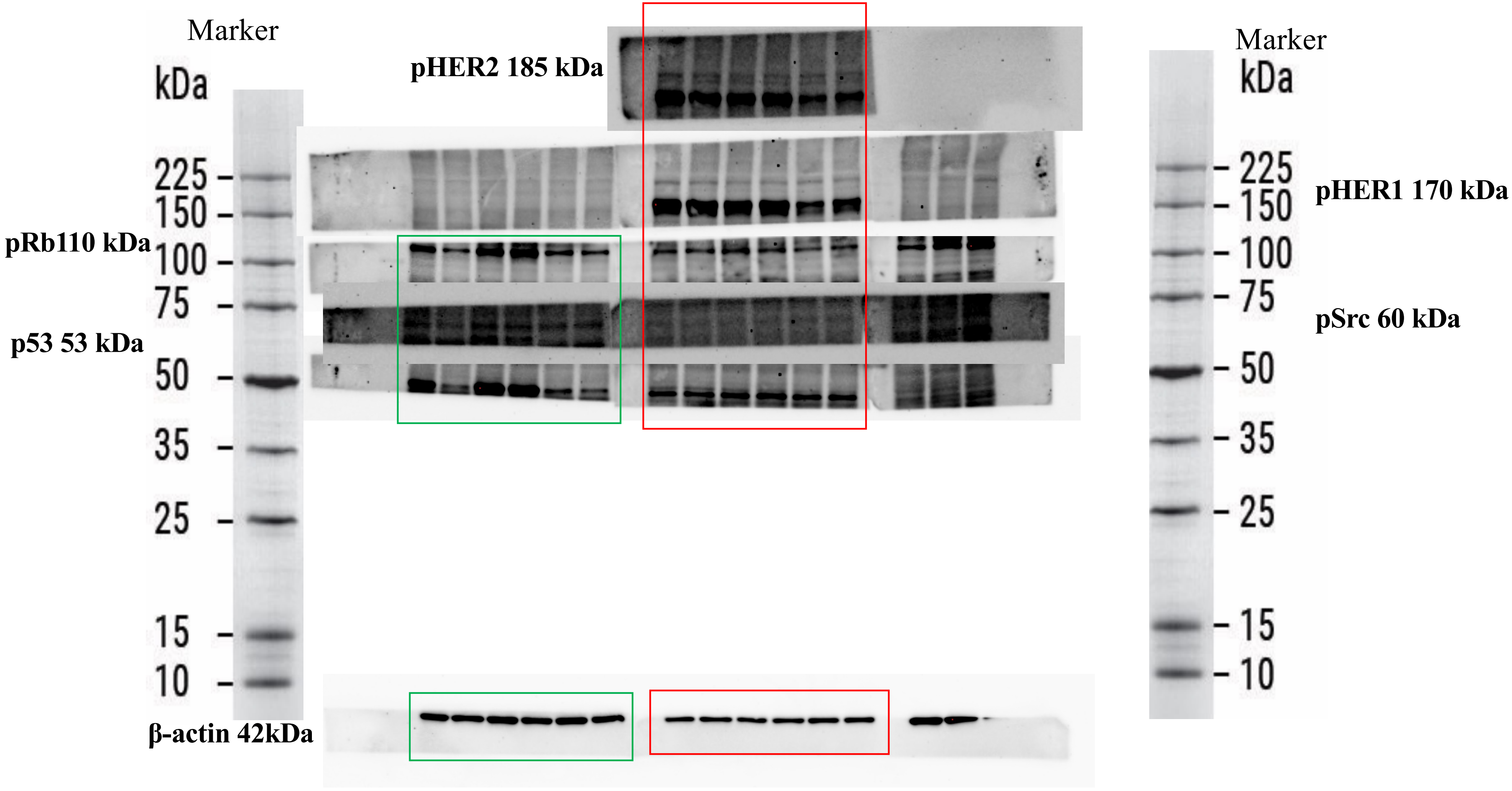

Original western blots from which Figure 8 (Panel A,B) derived: analysis of the expression levels of HER2, phospho-HER2, phospho-HER1, pRb protein (Ser 780), phosphorylated (p) Src, p53 and β-actin (loading control) in SK-BR-3 (red frame) or MDA-MB-231 cells (green frame). 20 µg of proteins/well were loaded.

SKBR3	Ctrl	Chaga 0.25 mg/ml	Cisplatin 1 μM	RJY13 0.01 μM	Cisplatin 1 μM + Chaga 0.25 mg/ml	RJY13 0.01 μM +Chaga 0.25mg/ml
pHER1	28045.912	28862.054	26667.922	28660.64	23709.418	25052.296
	27188.983	28026.761	25669.811	27546.347	22143.246	24131.539
	27996.619	29020.225	26764.861	28731.518	23882.539	25401.832
p53	24913.276	27753.468	25832.69	26958.569	24314.811	24179.518
	23416.518	26105.296	24551.811	25417.397	22305.64	21999.64
	24945.225	27106.054	25762.69	26621.933	23665.104	23480.518
pHER2	18858.569	17717.175	23313.296	19999.882	14043.397	17577.347
	18999.225	18642.832	23901.539	20607.589	14728.104	17592.347
	18005.569	15907.347	22694.175	18936.761	11989.276	16228.933
pRb	27345.326	26235.468	24407.518	25071.589	19954.418	22236.882
	27175.276	25925.468	24140.104	24796.882	19721.125	21904.418
	27021.276	25835.468	24063.811	24737.004	19613.589	21862.832
β-Actin	7235.548	8412.497	7239.79	7898.376	8236.719	8023.962
	6096.941	7046.669	6160.376	6872.376	7308.598	7084.598
	6984.426	8056.205	7031.497	7605.962	8024.841	7758.548
pHER1/β-actin	3.876	3.431	3.684	3.629	2.879	3.122
	4.459	3.977	4.167	4.008	3.030	3.406
	4.008	3.602	3.806	3.777	2.976	3.274
p53/β-Actin	3.443	3.299	3.568	3.413	2.952	3.013
	3.841	3.705	3.985	3.698	3.052	3.105
	3.572	3.365	3.664	3.500	2.949	3.026
pHER2/β-Actin	3.779	3.119	3.371	3.174	2.423	2.771
	4.457	3.679	3.919	3.608	2.698	3.092
	3.869	3.207	3.422	3.252	2.444	2.818
pRb/β-Actin	2.606	2.106	3.220	2.532	1.705	2.191
	3.116	2.646	3.880	2.999	2.015	2.483
	2.578	1.975	3.228	2.490	1.494	2.092

Corresponding to Figure 8A. Densitometry Readings/Intensity Ratio

MDA-MB 231	Ctrl	Chaga 0.25 mg/ml	Cisplatin 1 µM	RJY13 0.01 µM	Cisplatin 1 µM + Chaga 0.25 mg/ml	RJY13 0.01 µM +Chaga 0.25mg/ml
pRb	17205.326	13677.589	21599.589	24134.64	18854.418	14987.418
	15118.569	10946.054	19629.882	22260.225	16471.004	12341.004
	19139.569	15163.933	22818.175	25155.225	20310.004	16787.296
pSrc	15041.983	11671.861	14689.205	13901.276	12417.569	12785.761
	16883.983	12868.276	16293.033	15390.811	13622.811	13725.589
	23158.589	15363.761	23007.711	20878.246	16562.539	16545.296
p53	22156.184	18339.64	26544.154	27329.033	21149.569	20059.054
	20392.891	15811.225	24483.569	24973.569	18975.054	17699.64
	22792.012	19304.225	26664.74	27381.569	22455.225	21169.104
pErk	18218.355	21430.64	20875.619	21235.326	21052.69	22070.125
	16399.841	18745.175	18318.74	18776.619	18769.225	19543.64
	18529.941	22150.811	21549.619	21874.083	21936.397	22859.64
β-actin	11834.527	12331.669	13521.669	12266.012	13487.305	12389.397
	9612.184	9931.083	11258.376	9959.012	11178.719	9942.376
	12752.698	13763.79	14878.962	13622.255	15025.426	13815.205
pRb/β-Actin	1.454	1.109	1.597	1.968	1.398	1.210
	1.573	1.102	1.744	2.235	1.473	1.241
	1.501	1.102	1.534	1.847	1.352	1.215
pSrc/β-Actin	1.271	0.946	1.086	1.133	0.921	1.032
	1.757	1.296	1.447	1.545	1.219	1.381
	1.816	1.116	1.546	1.533	1.102	1.198
p53/β-Actin	1.872	1.487	1.963	2.228	1.568	1.619
	2.122	1.592	2.175	2.508	1.697	1.780
	1.787	1.403	1.792	2.010	1.494	1.532
pErK/β-Actin	1.539	1.738	1.544	1.731	1.561	1.781
	1.706	1.888	1.627	1.885	1.679	1.966
	1.453	1.609	1.448	1.606	1.460	1.655

Corresponding to Figure 8B. Densitometry Readings/Intensity Ratio
