# Supplementary material for: Chaga Mushroom Triterpenoids Inhibit Dihydrofolate Reductase and Act Synergistically with Conventional Therapies in Breast Cancer
Source: Biomolecules. 2024 Nov 17;14(11):1454. doi: 10.3390/biom14111454 (PMC11591880; doi:10.3390/biom14111454)
Supplement: Supplementary file 1 [file biomolecules-14-01454-s001.zip › biomolecules-3176010-supplementary figures and tables.pdf]

Table S1. Yields of Chaga extracts with ethyl acetate, methanol and water

Sample	Yield ethyl acetate (%)	Yields methanol (%)	Yields water (%)
Chaga non-digested	4.0	8.5	87.5
Chaga > 3.5 KDa	4.9	62.5	32.6
Chaga < 3.5 KDa	6.8	74.2	19.0

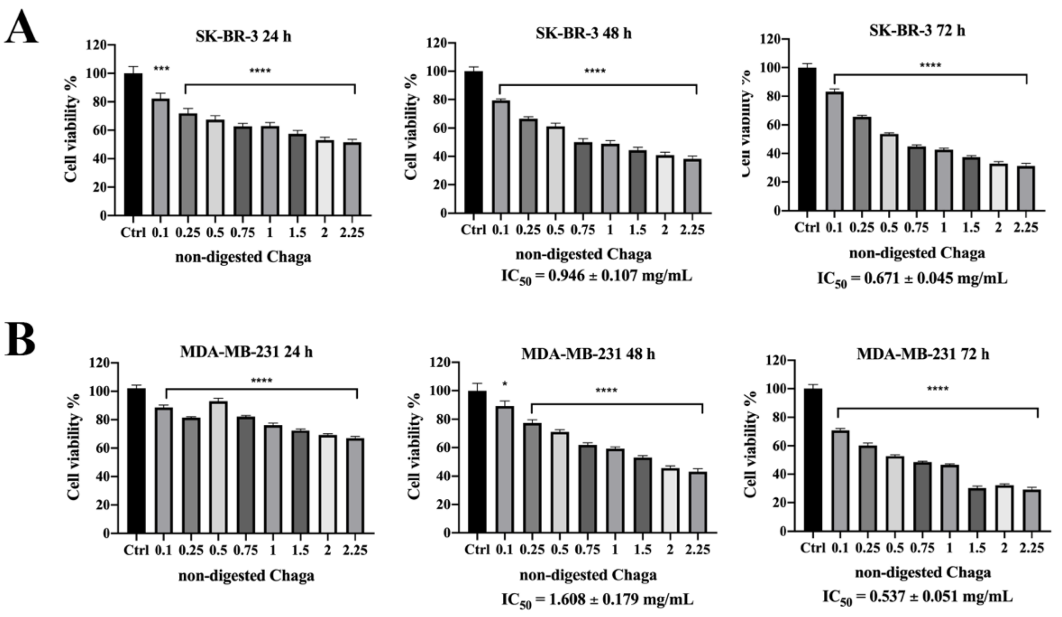

Supplementary Figure S1. Chaga water extract decreased breast cancer cell viability. (**A**) SK-BR-3 and (**B**) MDA-MB-231 cells were left untreated (control) or incubated for 24 h, 48 h or 72 h in the presence of increasing concentrations of Chaga water extract and cell viability was determined by MTT assay. The results are expressed as percentage of living cells with respect to control. Columns, mean of three separate experiments wherein each treatment was repeated in 6 wells; bars, SE. *p < 0.05, **p < 0.01, ***p < 0.001, ****p < 0.0001. One-way ANOVA followed by Dunnett's multiple comparisons test.

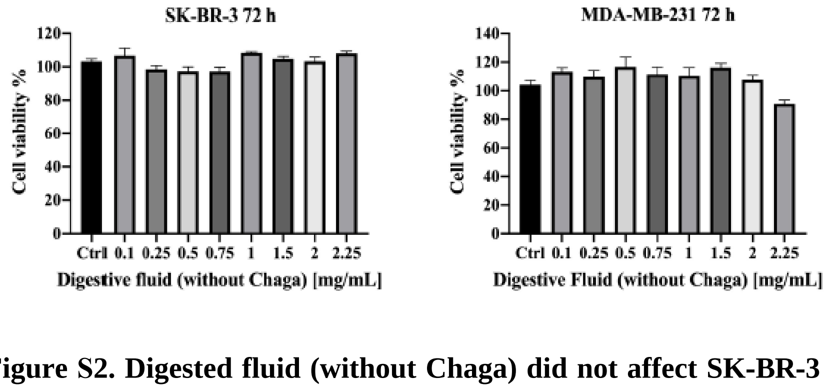

Supplementary Figure S2. Digested fluid (without Chaga) did not affect SK-BR-3 and MDA-MB-231 cell viability. Cells were left untreated (control) or incubated for 72 h in the presence of increasing concentrations of digested fluid (blank condition); cell viability was determined by MTT assay. The results are expressed as percentage of living cells with respect to control. Columns, mean of three separate experiments wherein each treatment was repeated in 6 wells; bars, SE.

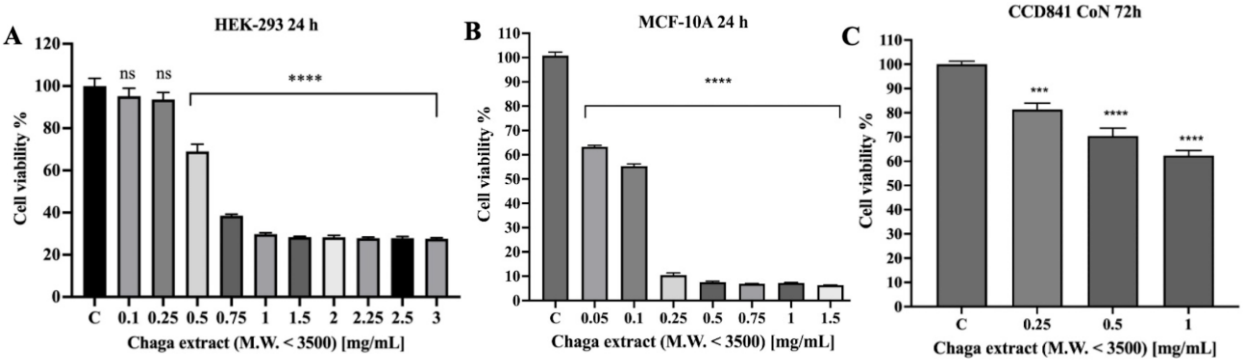

Supplementary Figure S3. Effect of digested Chaga extract on cell viability of non-cancerous cell lines. HEK-293 cells (A), MCF-10A (B) and CCD-841 CoN (C) were left untreated (control) or incubated at the indicated time in the presence of increasing concentrations of the low-molecular weight fraction (MW < 3500 Da) of digested Chaga water extract; cell viability was determined by MTT assay. The results are expressed as percentage of living cells with respect to control. Columns, mean of three separate experiments wherein each treatment was repeated in 6 wells; bars, SE. ***p <0.001, ****p < 0.0001. One-way ANOVA followed by Dunnett's multiple comparisons test.

Table S2. Digested Chaga extract (MW < 3500 Da) induced cell cycle arrest in the G0/G1 phase in SK-BR-3 cells. Cells were treated with Chaga extract at 1 mg/mL for 24 hours.

	**% G0/G1**	**% S**	**% G2/M**
Ctrl sample 1	58	23.3	18.8
Ctrl sample 2	54.4	27	18.8
Ctrl sample 3	57.1	23.6	19.2
Chaga 1 mg/ml sample 1	71.4	12.8	15.2
Chaga 1 mg/ml sample 2	69.5	12.5	17.6
Chaga 1 mg/ml sample 3	68.9	2.14	30

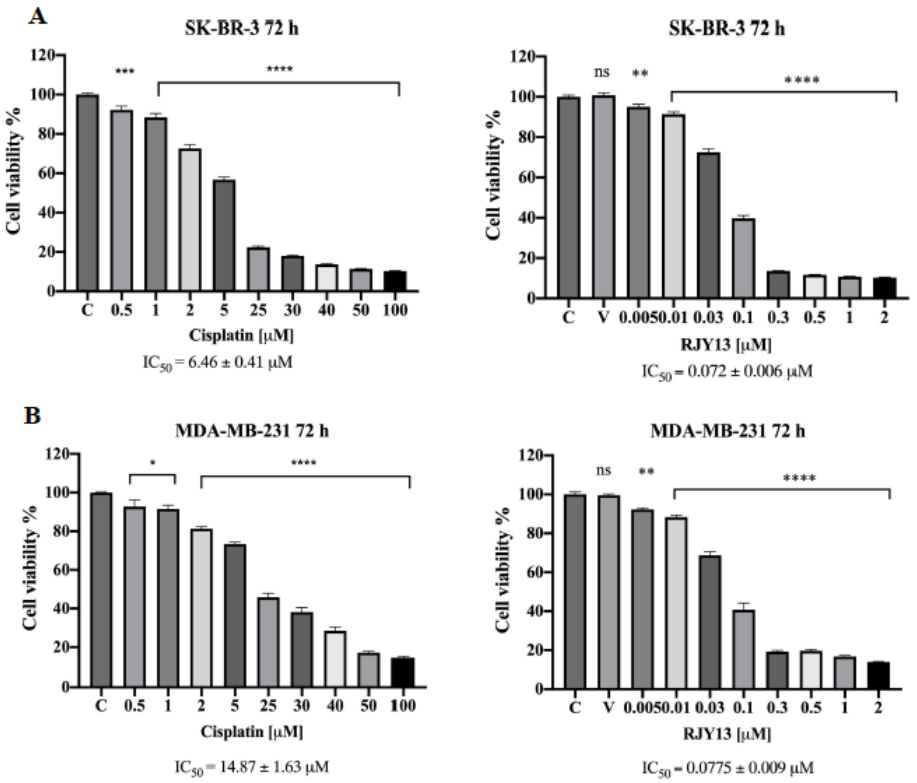

Supplementary Figure S4. Effect of cisplatin treatment on SK-BR-3 and MDA-MB-231 cell viability. SK-BR-3 cells (A) and MDA-MB-231 cells (B) were left untreated (control) or incubated for 72 h in the presence of increasing concentrations of cisplatin (left panel) or cisplatin derivative RJY13 (right panel) or its vehicle (V); cell viability was determined by MTT assay. The results are expressed as percentage of living cells with respect to control. Columns, mean of three separate experiments wherein each treatment was repeated in 6 wells; bars, SE. *p < 0.05, **p < 0.01, ***p <0.001, ****p < 0.0001. One-way ANOVA followed by Dunnett's multiple comparisons test.

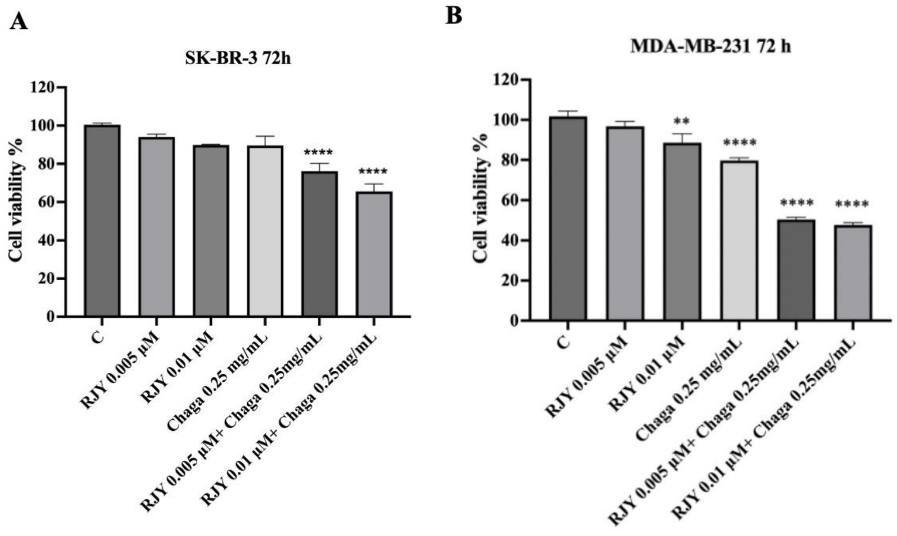

Supplementary Figure S5. Synergistic effect between Chaga and RJY13 (cisplatin derivative) treatment on SK-BR-3 and MDA-MB-231 cell viability. SK-BR-3 cells (A) and MDA-MB-231 cells (B) were left untreated (control) or incubated for 72 h in the presence of indicated concentrations of RJY13 or Chaga or their combination; cell viability was determined by MTT assay. The results are expressed as percentage of living cells with respect to control. Columns, mean of three separate experiments wherein each treatment was repeated in 6 wells; bars, SE. **p < 0.01, ****p < 0.0001. One-way ANOVA followed by Dunnett's multiple comparisons test.

Table S3. Drug interaction evaluated by Bliss Independence model

Figure 7B										
E(x,y)=Ex+Ey-(Ex*Ey)										
x: Chaga 0.25mg/mL		SK-BR-3(24h)	Ex	0.15						
			Concentration	Ey	Ex+Ey-(Ex*Ey)		E(x,y): Combination effect			
y: Trastuzumab		SK-BR-3 (24h)	0 µg/mL	0	0.15					
			1 µg/mL	0	0.15	=	0.15	Additive		
			2.5 µg/mL	0.05	0.1925	<	0.2	Synergistic		
			5 µg/mL	0.06	0.201	<	0.23	Synergistic		
			10 µg/mL	0.05	0.1925	<	0.24	Synergistic		
			15 µg/mL	0.05	0.1925	<	0.2	Synergistic		
			20 µg/mL	0.1	0.235	<	0.26	Synergistic		
			25 µg/mL	0.15	0.2775	>	0.24	Antagonistic		
			30 µg/mL	0.13	0.2605	>	0.24	Antagonistic		
Figure 7C										
							Ex+Ey-(Ex*Ey)		E(x,y): Combination	
x: Chaga 0.25mg/mL		SK-BR-3	72h	Ex	0.1					
y: Cisplatin		SK-BR-3	72h	Ey	0.5 µg/mL	0.08	0.172	<	0.22	Synergistic
					1 µg/mL	0.11	0.199	<	0.24	Synergistic
Figure 7D										
x: Chaga 0.25mg/mL		MDA-MB-231	72h	Ex	0.2					
y: Cisplatin		MDA-MB-231	72h	Ey	0.5 µg/mL	0	0.2	<	0.55	Synergistic
					1 µg/mL	0.05	0.24	<	0.6	Synergistic

The Bliss Independence model is based on the assumption that different agents act independently of each other in terms of their mode and the site of action. The basis of the Bliss Independence model is represented in the equation: E(x,y)= Ex + Ey - (Ex * Ey), where E is the fractional effect (between 0 and 1), and x and y are the doses (or concentrations) of drugs in the combination. If the combination effect is higher than the expected value from Equation, the interaction is synergistic, while if this effect is lower, the interaction is antagonistic. Otherwise, the effect is additive and there is no interaction. The Bliss Independence theory provides the statistical significance of drug interaction and can be easily applied to both in vitro experiments and clinical trials since the equation works even with data from single combination points (Goldoni M, Johansson C. A mathematical approach to study combined effects of toxicants in vitro: evaluation of the Bliss independence criterion and the Loewe additivity model. Toxicol In Vitro. 2007 Aug;21(5):759-69. doi: 10.1016/j.tiv.2007.03.003).
According to Bliss Independence model, we can observe a synergistic interaction when SK-BR-3 cell line was treated 24 hours with 0.25 mg/mL chaga extract plus incremental doses of trastuzumab from 2.5 µg/mL to 20 µg/mL (corresponding to Figure 7B). Moreover, we can observe a synergistic interaction when SK-BR-3 and MDA-MB-231 cell lines were treated 72 hours with 0.25 mg/mL chaga extract plus 0.5 µg/mL and 1 µg/mL Cisplatin, respectively (corresponding to Figure 7C and Figure 7D, respectively).

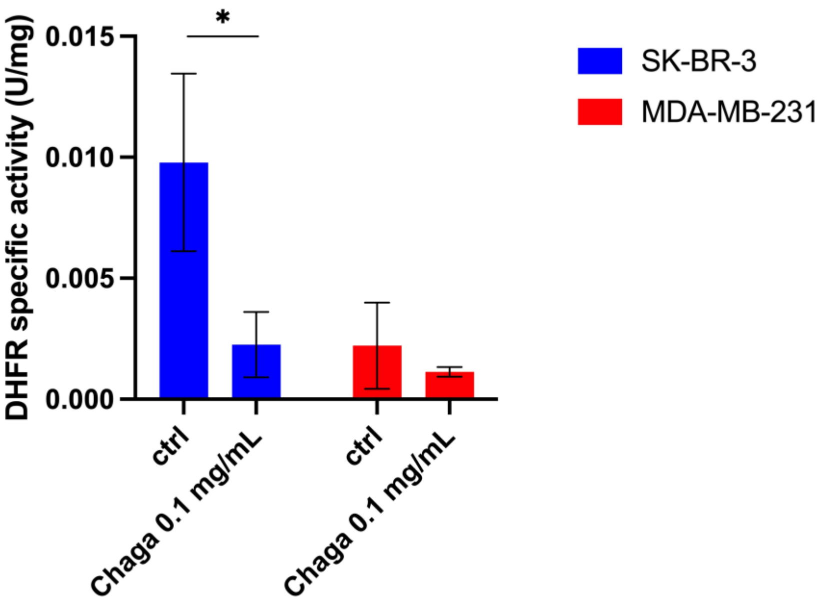

Supplementary Figure S6. Digested Chaga extract inhibited DHFR enzymatic activity in BC cells lysates. Untreated SKBR-3 (blue) and MDA-MB-231 (red) cells have been lysed after cell growth respectively for 6 and 4 hours and then DHFR activity was assayed after pre-incubation with 0.1 mg/mL of low-molecular weight fraction (MW < 3500 Da) of digested Chaga water extract. The results are expressed as the average of three replicates (± SE) of DHFR specific activity (U/mg), calculated after normalization with respect to the total protein content. One-way ANOVA followed by Tukey's multiple comparison test, *$p < 0.05$.
